# Numerical simulation and experimental evaluation of flow ripple characteristics of Truninger pump

**DOI:** 10.1038/s41598-022-15452-8

**Published:** 2022-07-04

**Authors:** Yundong Liang, Zongbin Chen, Jian Liao

**Affiliations:** 1 Insitute of Vibration and Noise, Naval University of Engineering, Wuhan, 430033 China; 2 State Key Laboratory of Ship Vibration and Noise, Wuhan, 430033 China

**Keywords:** Mechanical engineering, Mathematics and computing

## Abstract

Compared with involute internal gear pumps and gerotor pumps, lower flow ripple is the main advantage of Truninger pumps. Understanding the flow ripple mechanism and characteristics is of great significance to guide the design and manufacture of this type of pump. In this paper, the theoretical flow ripple and flow ripple rate expressions of the pump are derived based on the vector ray method, and the effects of variations of the design parameters of the pump on the theoretical flow ripple characteristics are studied. A three-dimensional numerical simulation model was established in Simerics-MP+ that accounted for the fluid properties and cavitation. All the geometric features, including unloading grooves, the oil distribution areas, the shapes of the suction and delivery passageways, and the axial and radial leakage gaps, were considered to achieve the highest accuracy in the prediction of flow ripple. Finally, a flow ripple test platform was built based on the secondary source method. The validity and accuracy of the model were verified by test results. The flow ripple characteristics under different working conditions were compared and analyzed. The following conclusions were obtained: (1) The smaller module, the larger addendum coefficient and the half angle of the tooth profile in the design process, the lower the pump speed during operation is beneficial to reduce the vibration and noise of this pump; (2) Flow ripple is the comprehensive result of the oil characteristics, internal leakage, and geometric characteristics through the comparisons of theoretical, simulation and experimental results; (3) The flow ripple amplitude and the ripple rate increased with the increase in the outlet pressure and the influence of the pump speed variations on the flow ripple characteristics is less than that of outlet pressure variations. The conclusions obtained in this paper will help designers understand the flow ripple mechanism, achieve low-noise pump designs, and optimize Truninger pumps.

## Introduction

The hydraulic pump is the core power component of a hydraulic system, with a high power density and reliability^1^. Due to the structure of the hydraulic pump itself and the compressibility of oil, the instantaneous flow rate at the outlet of the pump is not uniform, and periodic flow ripples appear^2^. Flow ripples are an inherent characteristic of hydraulic pumps and are the fundamental cause of fluid-borne noise in hydraulic systems. They interact with the impedance of the connected circuit, causing pressure ripples to form and spread across the whole system through the outlet, which leads to vibrations of pipes and the pump casing, mechanical and air-borne noise, and in severe cases, system instability and even resonance, causing serious damage to system components^3^. In general, the pump source flow ripple originates from the volume change of the variable pump chambers with the rotation angle of the pump components, which is called a theoretical flow ripple or kinematic flow ripple, and from other main factors including (1) internal leakage of the pump, (2) the geometry of the suction and discharge passageway, (3) the fluid properties (oil density, bulk modulus, and viscosity), and (4) cavitation effects in the fluid^4^.

Different types of pumps have different flow ripple characteristics^5^. The patent of a Truninger pump was first filed by Paul Truninger in 1970^6^. The tooth profile of its external gear and the internal gear ring are straight line and conjugate curve of the straight line respectively. Because of the different tooth shapes, the trapped oil volume is smaller, transmission is more stable, and the formation of output flow ripple is reduced compared to that of an involute internal gear pump and a gerotor pump. Due to the intellectual property protection of the tooth profile design of gear pairs, there are no openly available and complete standardized design systems at present. Therefore, research has mostly focused on tooth profile design theory^7,8^. The flow ripple characteristic analysis of Truninger pumps based on theory^9,10^ neglects many factors that influence the performance of the pump, and the research on the flow ripple characteristics of pumps lacks an effective numerical calculation model and specific experimental verification.

Different simulation approaches are used to reproduce the flow characteristics of the gear pump and the three-dimensional (3D) computational fluid dynamics (CFD) simulation approach is the best method to obtain detailed information about the flow field inside the pump^11^. Haworth et al.^12^ used a 3D CFD method to conduct simulations on the internal flow characteristics of external gear pumps for the first time. The product was optimized for noise reduction, and the volume efficiency was improved based on the results. Numerous studies based on professional software such as OpenFOAM^13,14^, ANSYS Fluent^15,16^, and ANSYS CFX^17,18^ have been performed on external gear pumps and gerotor pumps. Although most software includes a mesh deformation function, because the meshing region of gears involves large changes of the mesh topology, it is difficult to guarantee good grid quality during grid updating. Thus, optimal results cannot be obtained.

Simerics-MP+, formerly known as Pumplinx, is a software package that includes various types of gear pumps^19^. The mesh generator adopts a unique CAB algorithm in the computational domain to generate a cartesian grid, which is successful in filling tiny gaps in the computer-aided design (CAD) surface. The grid has a strong adaptive ability, fewer elements, convenient local encryption, high precision, and good convergence characteristics, which can solve the problem of inaccurate simulation results caused by the deterioration of the grid quality. The software has been proven to be suitable for various types of gear pumps, such as crescent pumps^9,20^, gerotor pumps^21^, and external gear pumps^22–24^.

To validate the simulation model, different methods have been developed to test the flow ripple of hydraulic pumps^25^. Of these methods, the secondary source method provided by Edge and Johnston et al. was formulated by International Organization for Standardization (ISO) as the standard method in 1996^26^. The secondary source method^3–5,27^ is based on wave transfer theory. Auxiliary pumps and valves with known frequency characteristics are used to change the terminal impedance of the test system where the tested pump is located, and the dynamic pressures at different positions of the connected pipes between the measured pump and auxiliary pumps and valves are synchronously tested by sensors to deduce the flow ripple of the pump source. The method is suitable for many types of positive displacement pumps, the testing frequency range is wide, the testing accuracy is high, and the test results have been verified by simulations^28,29^.

Compared with the previous work related to the Truninger pumps, some original work has been done in this paper. Firstly, A comprehensive 3D simulation model was developed in Simerics-MP+, design guidelines can be obtained from the simulation results. Then, the flow ripple test platform was built and improved to verify the simulation results effectively based on the secondary source method. Lastly, the effects of the outlet pressure and pump speed on the flow ripple characteristics were studied, which provides theoretical and technical support for low-noise design and optimization.

## Model description

**Figure 1:**
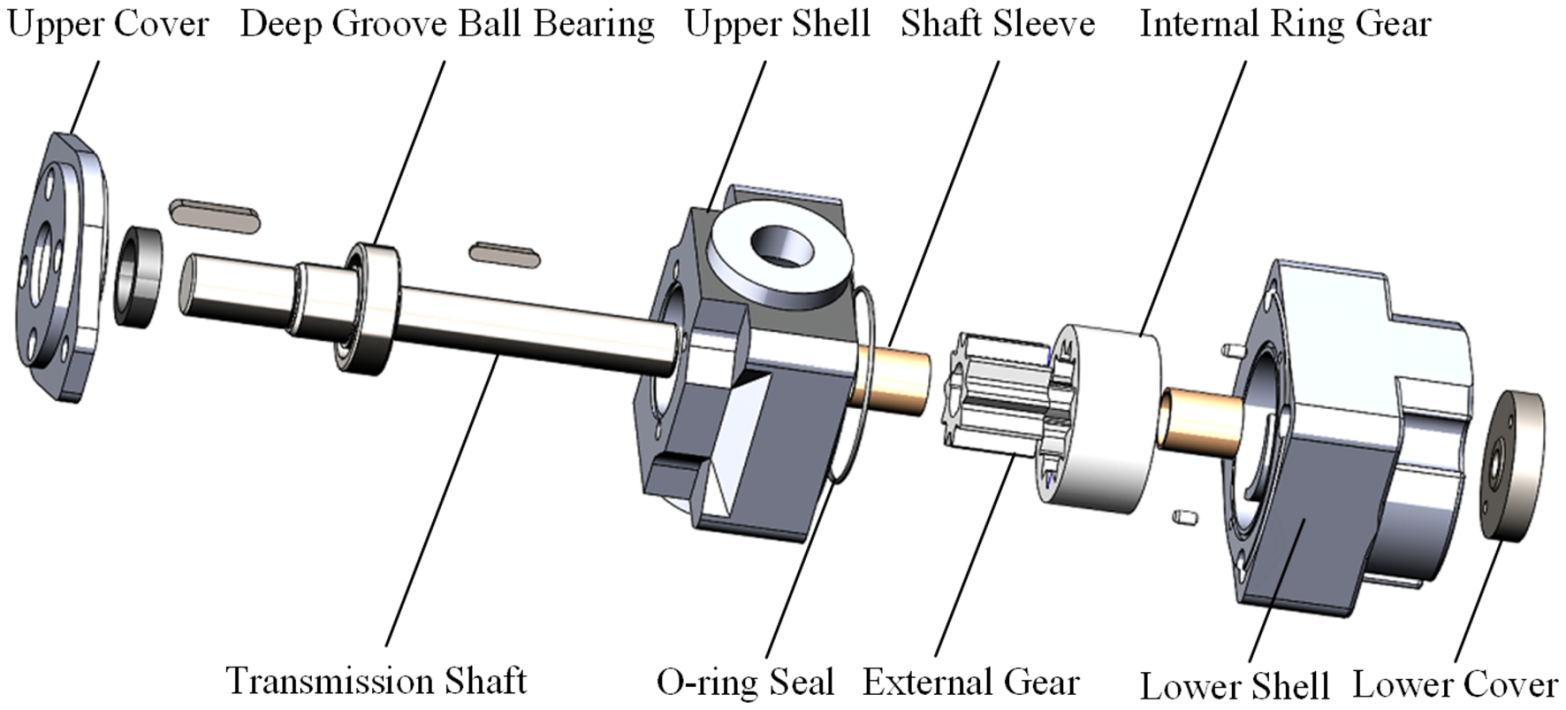
Structure of a Truninger pump.

**Figure 2:**
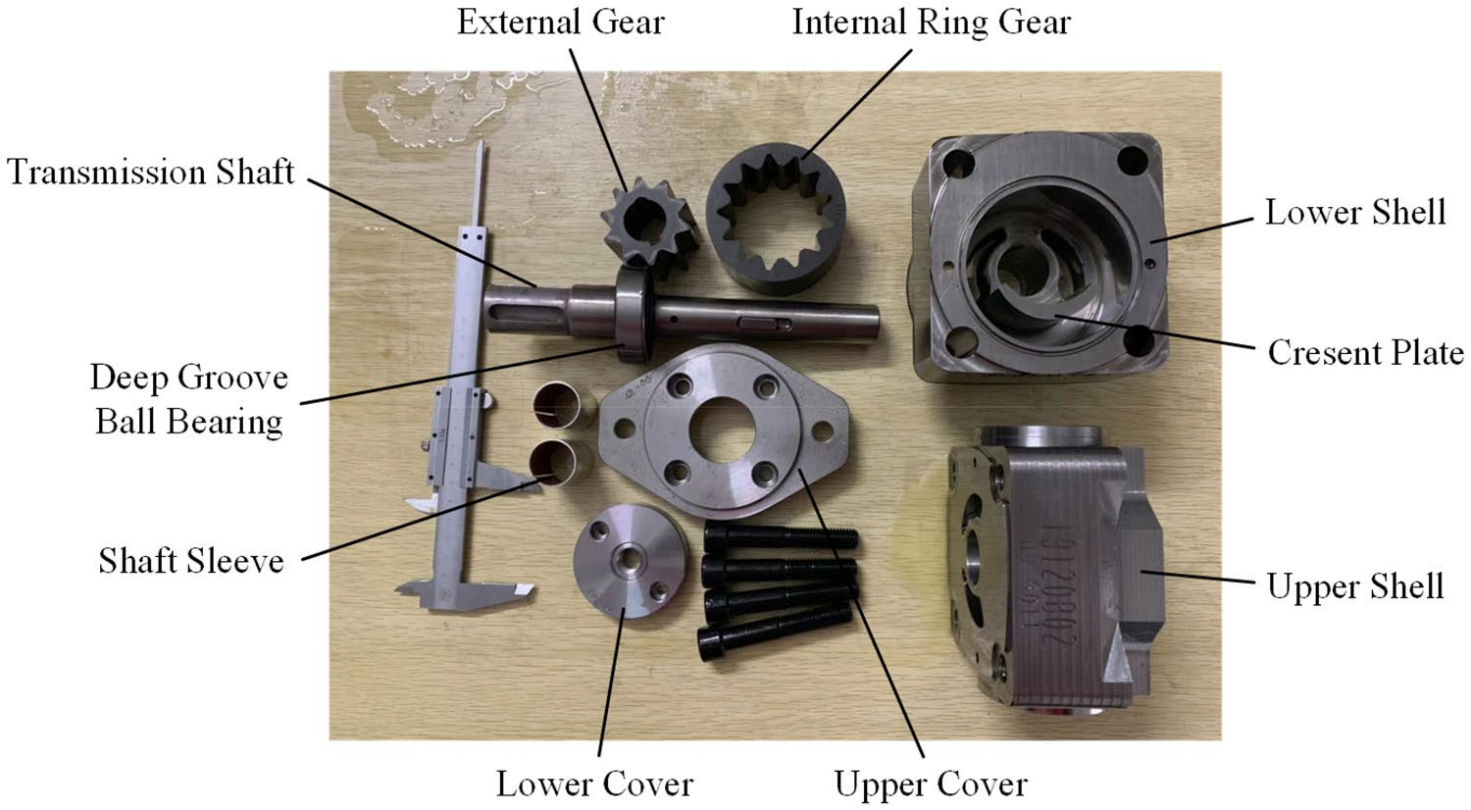
Photographs of the components of the pump.

**Figure 3:**
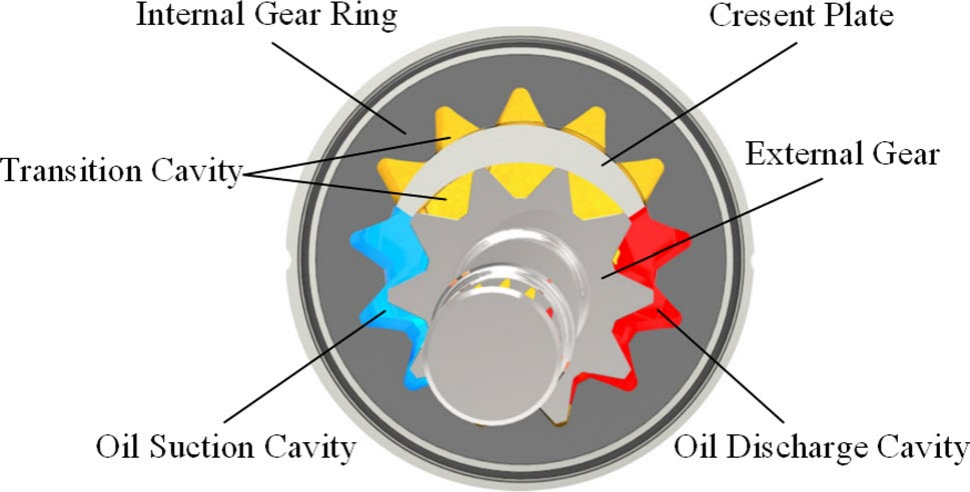
Schematic diagram of a Truninger pump.

The typical structure of a Truninger pump is shown in Fig. 1. The corresponding main characteristic parameters are listed in Table 1, and the reference model is designed and prototype is manufactured by our laboratory^8,30^. Its main components are an external gear, an internal gear ring, a transmission shaft, an upper shell and a lower shell. Photographs of each part of the pump are presented in Fig. 2. As shown in Fig. 3, the key components include an external gear with a straight-line tooth profile and an internal gear ring with a conjugated straight-line tooth profile, which are separated by a crescent plate. During operation, the inner and outer gears are separated at the oil suction cavity, and the cavity volume increases to form a vacuum to draw in oil. The oil between the gear teeth is transferred to the oil discharge cavity through the transition cavity formed by the gear pair and crescent plate. Then the inner and outer gears engage at the oil discharge cavity, and the cavity volume decreases to remove oil. An oil film of an appropriate thickness is formed between the sliding surface by the relative movement because of the existence of axial and radial clearance, which provides a dynamic seal in the pump, separating the oil suction cavity and oil discharge cavity.

**Table 1:** Main characteristic parameters.

Displacement (ml/r)	Rated speed (r/min)	Operating pressure (MPa)	Torque (N m)
25.1	1200r/min	12.5	50

## Modeling of theoretical flow ripple

### Geometric relations of external gear

**Figure 4:**
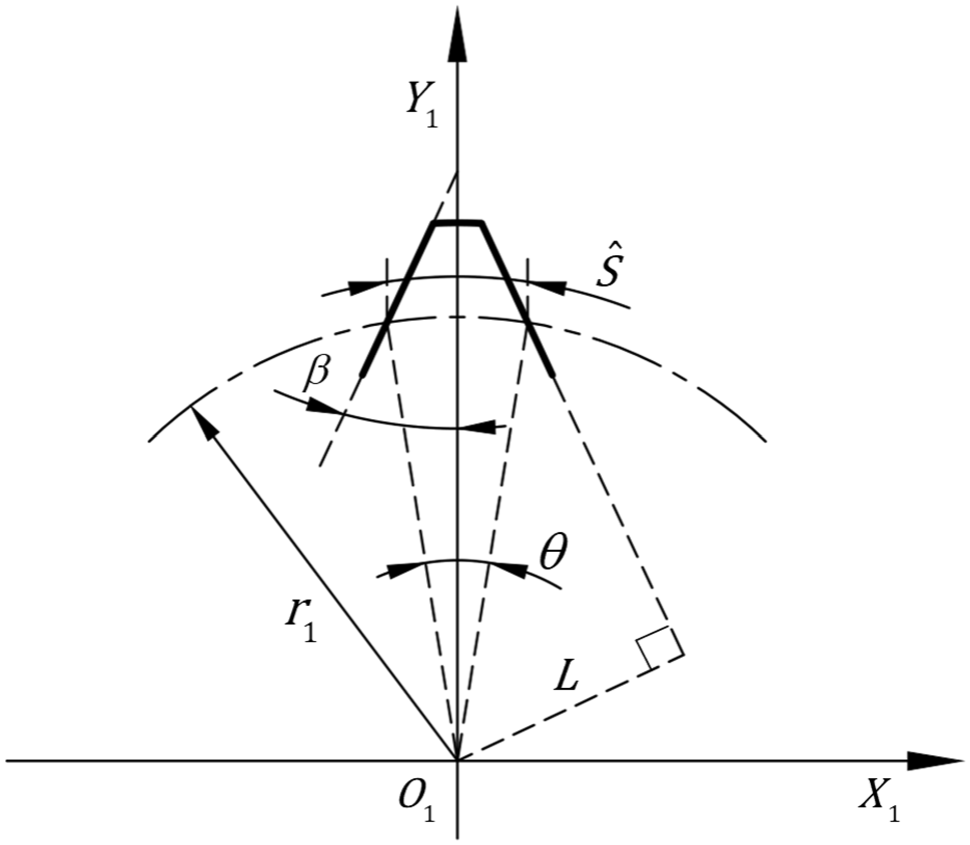
Geometric relations of the external gear.

The curve of the external gear tooth profile is a left–right symmetric straight line. The coordinate system $(O_{1} - X_{1} ,Y_{1} )$ is set up and the center of the external gear $O_{1}$ is the coordinate origin, $\theta = \frac{\pi }{{z_{1} }}$ is the central angle of the single tooth’s pitch circle, $\beta$ is the half angle of the tooth profile, $r_{1}$ is the pitch radius of the external gear, and $L$ is the perpendicular distance from the center of the external gear to one side of the tooth profile. Hence, the following relationship can be obtained from Fig. 4:

1 $$L = r_{1} \sin \left( {\frac{\theta }{2} + \beta } \right).$$

### Meshing relations of gear pair

**Figure 5:**
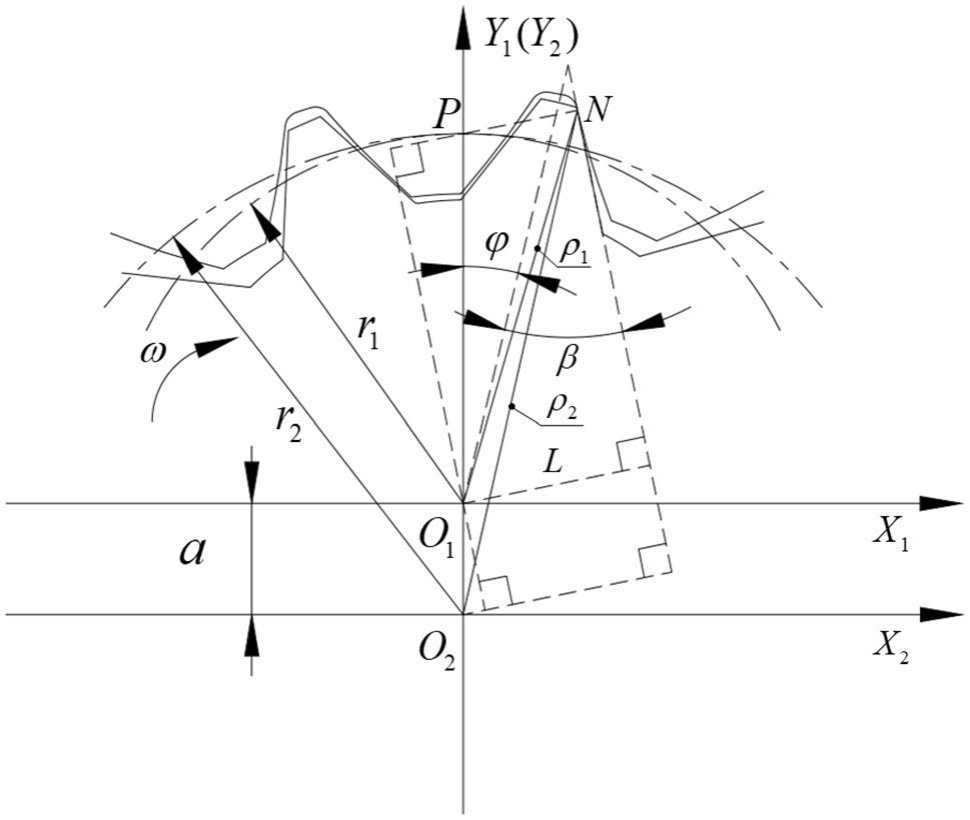
Meshing schematic diagram of the gear pair.

The schematic diagram of the meshing gear pair is shown in Fig. 5. Two fixed coordinate systems $(O_{1} - X_{1} ,Y_{1} )$ and $(O_{2} - X_{2} ,Y_{2} )$ were established at the center of the external gear $O_{1}$ and the internal gear ring $O_{2}$, respectively. At the starting position, the connection of the center of the external gear $O_{1}$ and the midpoint of the tooth tip coincided with the coordinate axis $Y_{1} (Y_{2} )$, where $N$ is the meshing point, $P$ is the pitch point, $\rho_{1}$ and $\rho_{2}$ are the distances from the external gear center and the internal gear ring center to the meshing point, respectively. Based on gear engagement theory^31^ and the geometric relationship in Fig. 5, the following equations for $\rho_{1}$ and $\rho_{2}$ can be obtained:

2 $$\rho_{1}^{2} = L^{2} + r_{1}^{2} \cos^{2} \left( {\beta - \varphi } \right),$$

3 $$\rho_{2}^{2} = \left[ {L + a\sin \left( {\beta - \varphi } \right)} \right]^{2} + r_{2}^{2} \cos^{2} \left( {\beta - \varphi } \right),$$

where $\varphi$ is the rotation angle of the external gear, $a$ is the center distance, $r_{1}$ and $r_{2}$ are the pitch radius of the external gear and the internal gear ring, respectively.

### Theoretical flow ripple characteristics

**Figure 6:**
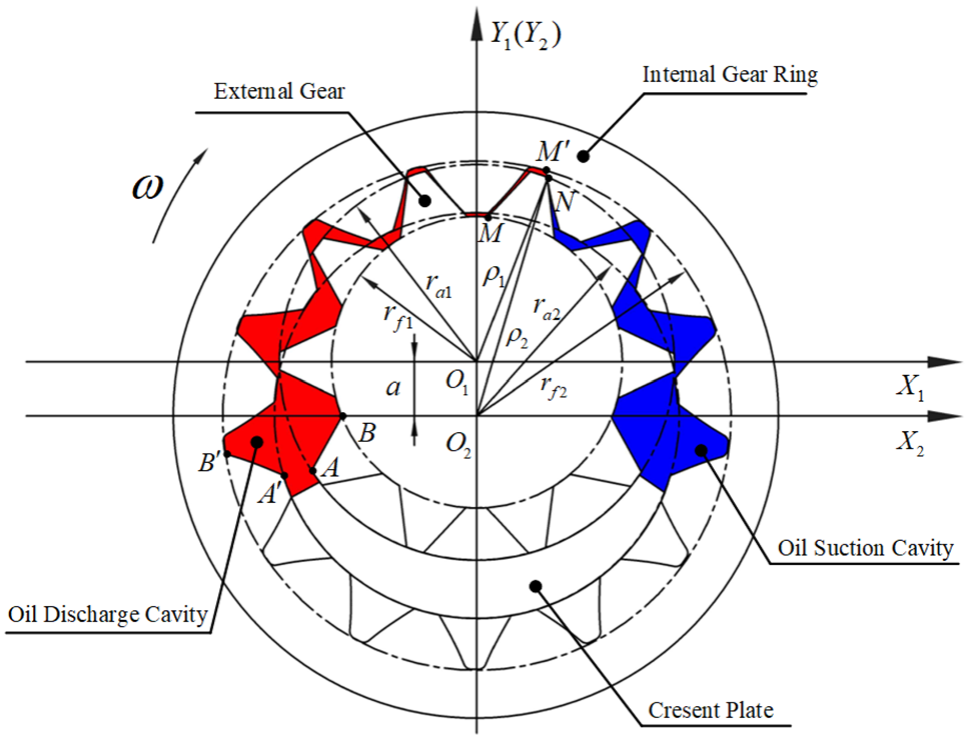
Schematic diagram of the vector ray method.

The vector ray method^32,33^ is a common method to analyze the theoretical flow ripple of different types of pumps. As shown in Fig. 6, the oil discharge cavity is sealed by the tooth profile of the external gear $A - B - M - N$, the tooth profile of the internal gear ring $A^{\prime} - B^{\prime} - M^{\prime} - N$, and the crescent plate. When the external gear rotates clockwise, the tooth profiles $A - B$ and $A^{\prime} - B^{\prime}$ squeeze the volume in the oil discharge cavity, making the cavity smaller. The tooth profiles $M - N$ and $M^{\prime} - N$ gradually move out of the meshing area, so that the volume of the cavity increases. The other tooth profiles that are completely surrounded by high-pressure oil in the oil discharge cavity have no effect on the volume change during rotation. Figure 6 shows that the expanded volume by rotation of the tooth profiles $M - N$ and $M^{\prime} - N$ is less than the compressed volume by rotation of the tooth profiles $A - B$ and $A^{\prime} - B^{\prime}$ in the meshing process, resulting in a continuous decrease in the volume of the oil discharge cavity. Similarly, the volume of the oil suction cavity formed by the tooth profiles of the internal gear ring, the external gear on the right side of the meshing point $N$, and the crescent plate increased continuously, forming a local negative pressure to draw oil into the pump.

Based on the above description, a mathematical model is established, and the following basic assumptions are made:

1. The influence of the oil characteristics are not considered.
2. The influence of the delivery pressure and internal leakage of the pump are not considered.
3. The influence of geometric features other than those of the rotor are not considered.
4. The coincidence degree of the gear pair is assumed to be 1.

The external gear rotates at an angular speed of $\omega$, and the rotation angles of the external gear and the internal gear ring are $\Delta \varphi_{1}$ and $\Delta \varphi_{2}$, respectively. And $i_{12} = {{r_{2} } \mathord{\left/ {\vphantom {{r_{2} } {r_{1} }}} \right. \kern-\nulldelimiterspace} {r_{1} }} = {{\Delta \varphi_{1} } \mathord{\left/ {\vphantom {{\Delta \varphi_{1} } {\Delta \varphi_{2} }}} \right. \kern-\nulldelimiterspace} {\Delta \varphi_{2} }}$ is the transmission ratio according to the principle of gear meshing. Based on Fig. 6, the reduction of the volume of the oil discharge cavity by the rotation of tooth profiles $A - B$ and $A^{\prime} - B^{\prime}$ can be expressed as follows:

4 $$\Delta V_{1} = b\left( {\frac{{r_{a1}^{2} - r_{f1}^{2} }}{2}\Delta \varphi_{1} + \frac{{r_{f2}^{2} - r_{a2}^{2} }}{2}\Delta \varphi_{2} } \right).$$

Similarly, the rotation of tooth profiles $M - N$ and $M^{\prime} - N$ enlarges the volume of the oil discharge cavity, and the volume increase can be expressed as follows:

5 $$\Delta V_{2} = b\left( {\frac{{\rho_{1}^{2} - r_{f1}^{2} }}{2}\Delta \varphi_{1} + \frac{{r_{f2}^{2} - \rho_{2}^{2} }}{2}\Delta \varphi_{2} } \right).$$

The volume change of the oil discharge cavity can be obtained by subtracting Eqs. (4) and (5):

6 $$\Delta V = \Delta V_{1} - \Delta V_{2} = b\left( {\frac{{r_{a1}^{2} - \rho_{1}^{2} }}{2}\Delta \varphi_{1} + \frac{{\rho_{2}^{2} - r_{a2}^{2} }}{2}\Delta \varphi_{2} } \right),$$

where $b$ is the tooth width, $r_{a1}$ and $r_{f1}$ are the radii of the addendum circle and the dedendum circle of the external gear, respectively, $r_{a2}$ and $r_{f2}$ are the radii of the addendum circle and the dedendum circle of the internal gear ring, respectively.

Because the position of the meshing point $N$ changes periodically with time, $\rho_{1}$ and $\rho_{2}$ change periodically with the position of the meshing point, which leads to periodic changes of $\Delta V$, making the output flow of the pump uneven. Therefore, according to this theoretical analysis, the instantaneous output flow of the pump must exhibit pulsating characteristics, which is the root cause of flow ripple.

The theoretical flow ripple ($q_{sh}$) of the pump can be obtained by taking the derivative of both sides of Eq. (6) with respect to time:

7 $$q_{sh} = \frac{\Delta V}{{\Delta t}} = \frac{b\omega }{2}\left[ {\left( {r_{a1}^{2} - \rho_{1}^{2} } \right) + \left( {\rho_{2}^{2} - r_{a2}^{2} } \right)\frac{{r_{1} }}{{r_{2} }}} \right]$$

By substituting the Eqs. (2) and (3) into Eq. (7), the theoretical flow ripple based on the rotation angle change of the external gear can be obtained:

8 $$q_{sh} { = }\frac{b\omega }{2}\left[ {\left( {r_{1}^{2} + i_{12}^{ - 1} a^{2} - i_{12} r_{1}^{2} } \right)\sin^{2} \left( {\beta - \varphi } \right) + 2i_{12}^{ - 1} aL\sin \left( {\beta - \varphi } \right) + } \right.\left. {r_{a1}^{2} + i_{12} r_{1}^{2} + i_{12}^{ - 1} L^{2} - i_{12}^{ - 1} r_{a2}^{2} - r_{1}^{2} - L^{2} } \right].$$

The oil discharge volume of a pair of teeth meshing is as follows:

9 $$q_{0} = \int\limits_{\varphi }^{{\varphi + \frac{2\pi }{{z_{1} }}}} {q_{sh} dt} .$$

The number of the external gear teeth involved in the meshing is $z_{1}$ when each gear pair rotates once, so the theoretical displacement of the gear pump can be obtained as follows:

10 $$\begin{aligned} & q = q_{0} z_{1} { = }\frac{{bz_{1} }}{2}\left\{ {\left( {r_{1}^{2} + i_{12}^{ - 1} a^{2} - i_{12} r_{1}^{2} } \right)\left[ {\frac{\pi }{{z_{1} }} - \frac{1}{4}\sin 2\left( {\varphi + \frac{2\pi }{{z_{1} }} - \beta } \right) + \frac{1}{4}\sin 2\left( {\varphi - \beta } \right)} \right]{ + }2i_{12}^{ - 1} aL} \right. \\ & \times \;\left. {\left[ {\cos \left( {\varphi + \frac{2\pi }{{z_{1} }} - \beta } \right) - \cos \left( {\varphi - \beta } \right)} \right]{ + }\left( {r_{a1}^{2} + i_{12} r_{1}^{2} + i_{12}^{ - 1} L^{2} - i_{12}^{ - 1} r_{a2}^{2} - r_{1}^{2} - L^{2} } \right)\frac{2\pi }{{z_{1} }}} \right\}. \\ \end{aligned}$$

The hydraulic pump flow ripple characteristics are generally evaluated by the flow ripple amplitude and the flow ripple rate^34^, which are defined as follows:

11 $$q_{a} = q_{sh\max } - q_{sh\min } ,$$

12 $$\delta_{q} = \frac{{q_{sh\max } - q_{sh\min } }}{{q_{t} }}.$$

$q_{t}$ is the average flow rate of the pump, which refers to the volume of oil discharged by the pump per unit time:

13 $$q_{t} = qn,$$

where $n$ is the pump speed.

### Influences of parameters on flow ripple characteristics

The geometric parameters of the gear pair are shown in Table 2. The speed of the pump was set to 1200 r/min as an example. The relation between rotation angle and rotation time of external gear is as follows:

14 $$\varphi = \omega t = 2\pi nt$$

**Table 2:** Parameters of the gear pair.

Parameters	Symbol	Value
Module	$m$	4
Number of external gear teeth	$z_{1}$	10
Number of internal gear ring teeth	$z_{2}$	13
Addendum coefficient	$h_{a}^{ * }$	0.7
Dedendum coefficient	$h_{d}^{ * }$	0.8
Tooth thickness central angle	$\theta$	18°
Half angle of the tooth profile of external gear	$\beta$	25.5°
Tooth width	$b$	34 mm
Pitch radius of the external gear	$r_{1} = mz_{1} /2$	20 mm
Pitch radius of the internal gear ring	$r_{2} = mz_{2} /2$	26 mm
Addendum radius of the external gear	$r_{a1} = r_{1} + h_{a}^{ * } m$	22.8 mm
Dedendum radius of the internal gear ring	$r_{f1} = r_{1} - h_{d}^{ * } m$	16.8 mm

**Figure 7:**
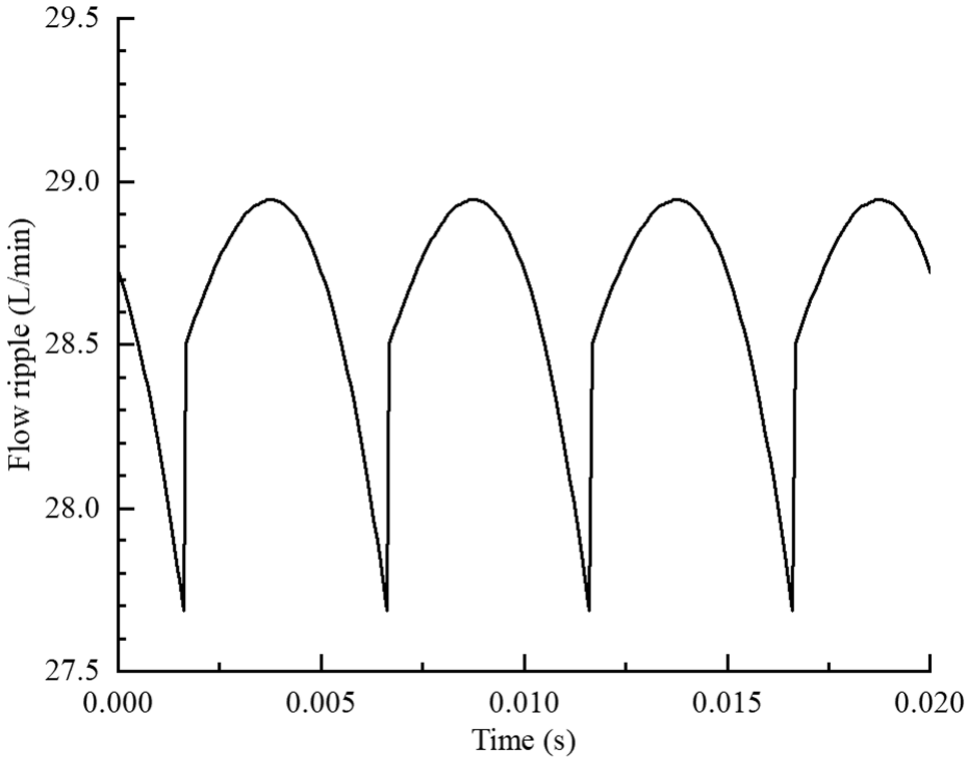
Theoretical flow ripple ($n$ = 1200 r/min).

The Eq. (14) and the above parameters were substituted into Eq. (8), thereby, the theoretical flow ripple curves corresponding to the rotation time of external gear were obtained by programming in MATLAB and the results are shown in Fig. 7.

As shown in Fig. 7, the theoretical flow ripple curve of the gear pump exhibited a periodic toothlike distribution, and the variation period was 0.005 s. The maximum value of the flow ripple curve was 28.94 L/min, the minimum value on the flow ripple curve was 27.62 L/min, and the average flow rate was 28.63 L/min. The flow ripple amplitude $q_{a}$ was 1.32 L/min, and the flow ripple rate $\delta_{q}$ was 4.61%.

In general, $z_{1}$, $z_{2}$ are defined design parameters^7^. Therefore, the main parameters affecting the flow characteristics of the pump are $r_{1}$, $r_{a1}$, $r_{a2}$, $\beta$, and $n$. When ${\text{z}}_{1}$ and $z_{2}$ are specified, $r_{1}$, $r_{a1}$, and $r_{a2}$ are completely determined by $m$ and $h_{a}^{ * }$ according to Table 2, so the influence of $m$, $h_{a}^{ * }$, $\beta$, and $n$ on the average flow rate and flow ripple characteristics of the pump are programmed in MATLAB based on the Eqs. (11–13).

**Figure 8:**
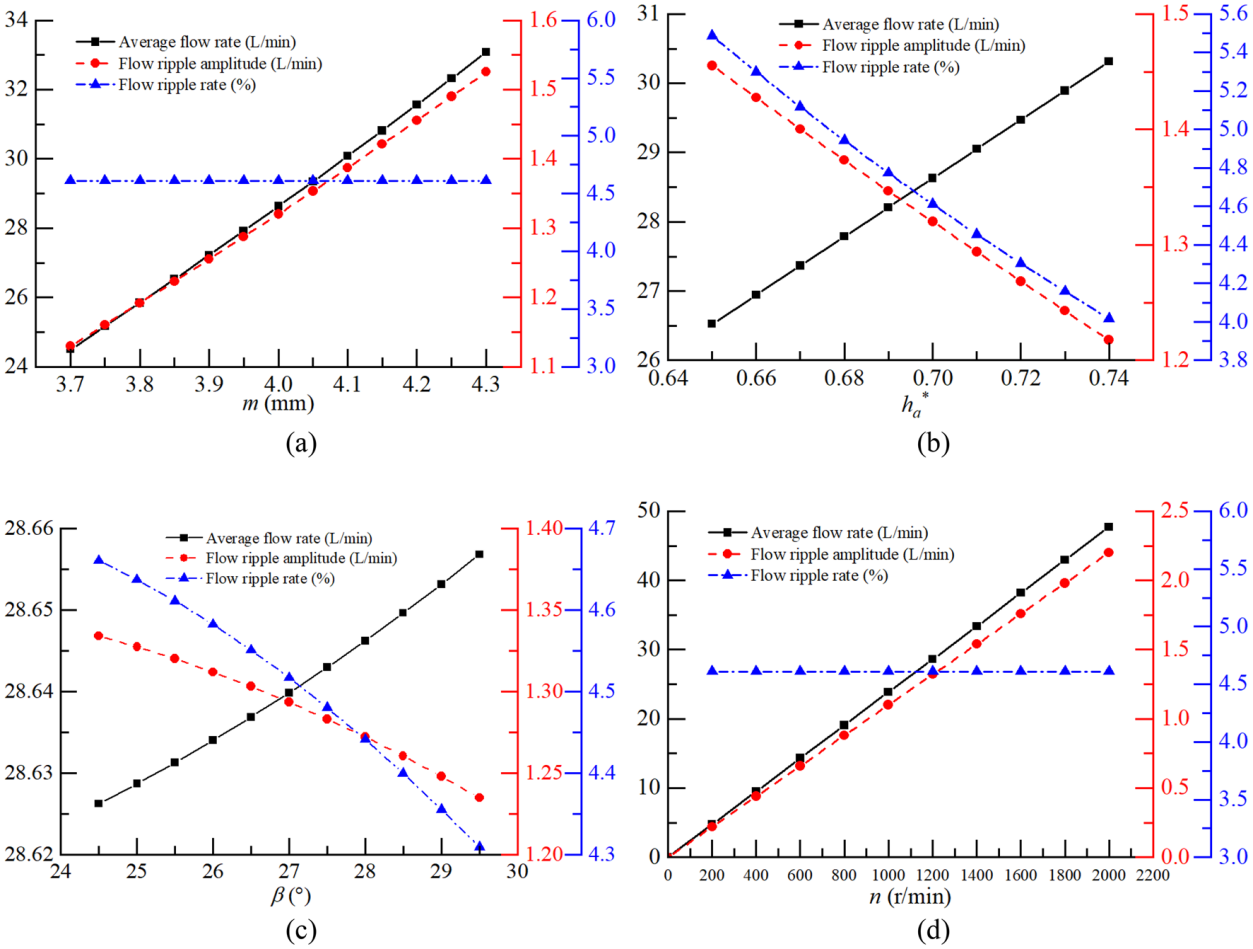
Influence of parameters on the average flow rate and flow ripple characteristics: (**a**) module, (**b**) addendum coefficient, (**c**) half angle of the tooth profile, and (**d**) pump speed.

The value ranges of the design variables were determined according to relevant design theory^7^. The specific results were as follows. The module $m$ was 3.7–4.3 mm, the addendum coefficient $h_{a}^{ * }$ was 0.65–0.74, the half angle of the tooth profile $\beta$ was 24.5–29.5°, and the pump speed $n$ was 0–2000 r/min. The calculated results are shown in Fig. 8.

The following inferences can be drawn from Fig. 8:

1. When designing a gear pump with a specified average flow rate, the module should be as small as possible and the addendum coefficient and the half angle of the tooth profile should be as large as possible because the flow ripple rate and the flow ripple amplitude are lower, so that the vibrations and noise level of the designed gear pump are lower.
2. The flow ripple rate is unaffected by the pump speed and the speed should be reduced as much as possible to reduce the flow ripple amplitude when the pump speed meets the flow requirements of the hydraulic system. The vibrations and noise of the system under this working condition are minimal.

## Numerical methods

### Computational model

**Figure 9:**
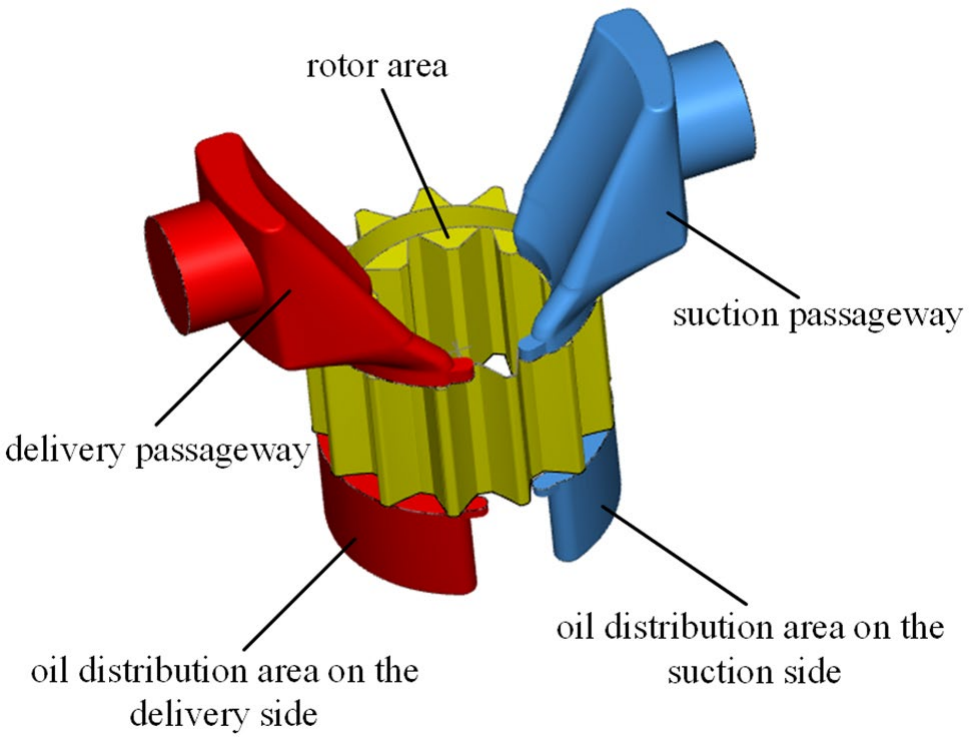
Internal fluid domain of Truninger pump.

**Figure 10:**
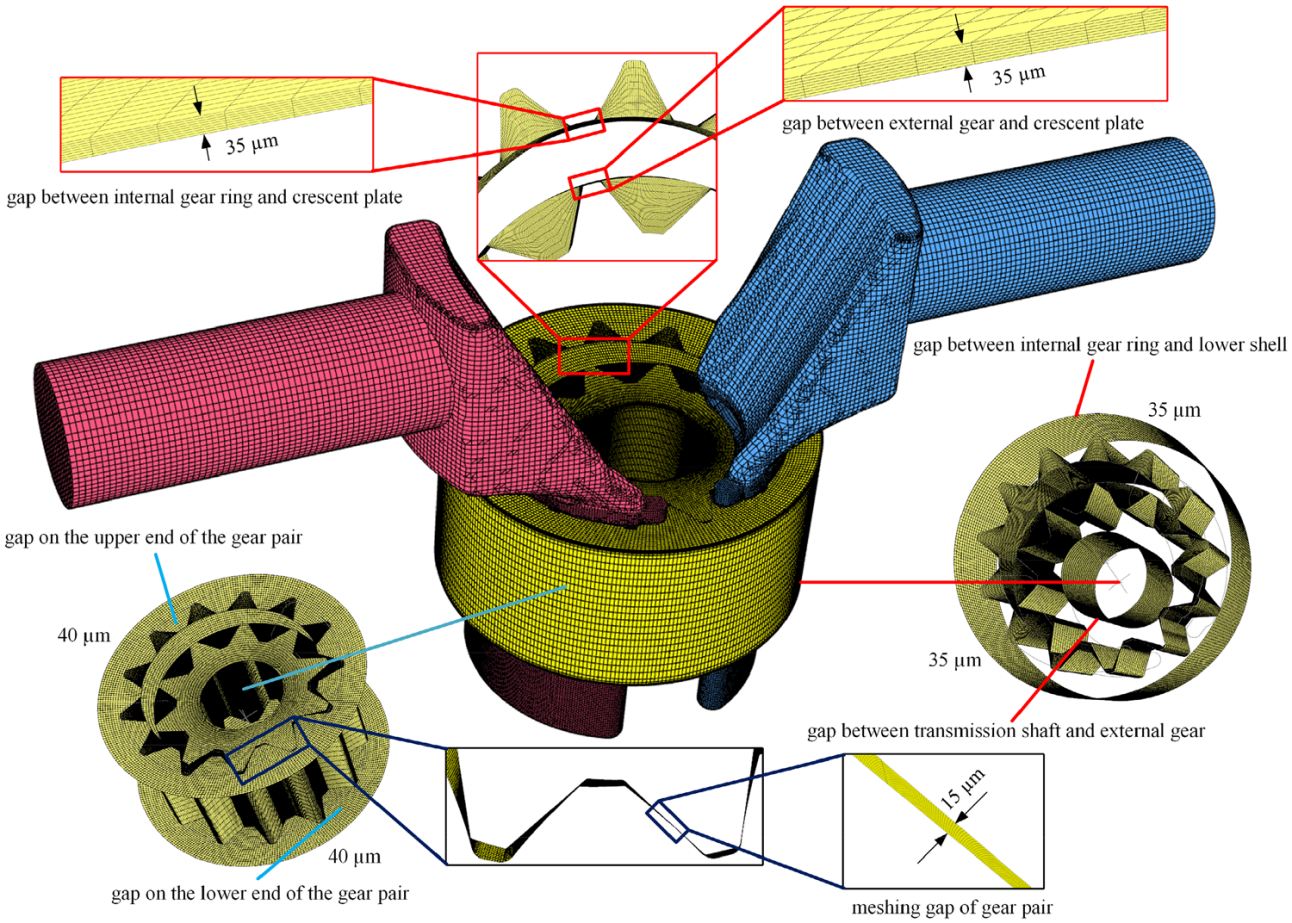
Grid model of Truninger pump.

The model of the Truninger pump is shown in Fig. 1. Simerics-MP + directly modeled the internal fluid domain of the pump extracted from the CAD software. As shown in Fig. 9, the fluid domain was mainly composed of five parts: a suction passageway, delivery passageway, oil distribution area on the suction side, oil distribution area on the delivery side, and rotor area, which were meshed and interacted in the software. In the working process of a gear pump, the surfaces of each component are separated by a gap containing an oil film with a certain thickness, forming a friction pair to achieve lubrication and prevent the scratching of the surfaces of the components. Thus, the friction pair surface of the model included a corresponding oil film grid to simulate the realistic conditions of a pump. Based on engineering experience^35^, the radial oil film thickness was 35 µm, and it filled included the following gaps: (1) the gap between the internal gear ring and the crescent plate, (2) the gap between the external gear and the crescent plate, (3) the gap between the transmission shaft and the external gear, and (4) the gap between the internal gear ring and the lower shell. The axial oil film thickness was 40 µm, which was on the upper and lower ends of the gear pair. The outer diameter of the oil film was the diameter of the internal gear ring, and the inner diameter was the diameter of the transmission shaft. The initial meshing gap of the gear pair was 15 µm. The suction and delivery passageways were appropriately extended to prevent the influence of backflow on the simulation results and accelerate the convergence of the results. The actual generated grid model of the Truninger pump is shown in Fig. 10.

### Governing equations

Simerics-MP + solves the fundamental equations of conservation of mass and momentum^19^. The following are its basic integral forms:

15 $$\frac{\partial }{\partial t}\int\limits_{\Omega } {\rho d\Omega } + \int\limits_{\sigma } {\rho \left( {\overrightarrow {\nu } - \overrightarrow {{\nu_{\sigma } }} } \right) \cdot \overrightarrow {n} d\sigma } = 0,$$

16 $$\frac{\partial }{\partial t}\int\limits_{\Omega } {p\overrightarrow {\nu } d\Omega } + \int\limits_{\sigma } {\rho \left( {\left( {\overrightarrow {\nu } - \overrightarrow {{\nu_{\sigma } }} } \right) \cdot \overrightarrow {n} } \right)\overrightarrow {\nu } d\sigma = } \int\limits_{\sigma } {\widetilde{\tau } \cdot \overrightarrow {n} d\sigma } - \int\limits_{\sigma } {p\overrightarrow {n} d\sigma } + \int\limits_{\Omega } {\overrightarrow {f} d\Omega } ,$$

#### Turbulence model

Mature turbulence models, such as the standard $k - \varepsilon$ model^36^ and renormalization group (RNG) $k - \varepsilon$ model^37^, have been applied for modeling turbulent systems and proved to be suitable for practical engineering applications^20–24^. The RNG $k - \varepsilon$ model can deal with flow with high strain rates and large curved streamlines better, and it avoids the distortion caused by the standard $k - \varepsilon$ model when applied to a flow with a strong swirling and curved wall flow. Therefore, the RNG $k - \varepsilon$ model was used in this study. The integral form of the RNG $k - \varepsilon$ model can be written as follows:

17 $$\frac{\partial }{\partial t}\int\limits_{\Omega } {\rho k \cdot d\Omega } + \int\limits_{\sigma } {\rho \left( {\left( {\overrightarrow {\nu } - \overrightarrow {{\nu_{\sigma } }} } \right) \cdot \overrightarrow {n} } \right)kd\sigma = } \int\limits_{\sigma } {\left( {\mu + \frac{{\mu_{t} }}{{\sigma_{k} }}} \right)(\nabla k \cdot \overrightarrow {n} )d\sigma } + \int\limits_{\Omega } {(G_{t} - \rho \varepsilon )d\Omega } ,$$

18 $$\frac{\partial }{\partial t}\int\limits_{\Omega } {\rho \varepsilon \cdot d\Omega } + \int\limits_{\sigma } {\rho \left( {\left( {\overrightarrow {\nu } - \overrightarrow {{\nu_{\sigma } }} } \right) \cdot \overrightarrow {n} } \right)\varepsilon d\sigma = } \int\limits_{\sigma } {\left( {\mu + \frac{{\mu_{t} }}{{\sigma_{\varepsilon } }}} \right)(\nabla \varepsilon \cdot \overrightarrow {n} )d\sigma } + \int\limits_{\Omega } {(c_{1} G_{t} \frac{\varepsilon }{k} - c_{2} (RNG) \cdot \rho \frac{{\varepsilon^{2} }}{k})d\Omega } ,$$

19 $$c_{2} (RNG) = c_{2} + \frac{{C_{\mu } \eta^{3} (1 - {\raise0.7ex\hbox{$\eta $} \!\mathord{\left/ {\vphantom {\eta {\eta_{0} }}}\right.\kern-\nulldelimiterspace} \!\lower0.7ex\hbox{${\eta_{0} }$}})}}{{1 + \beta_{0} \eta^{3} }},$$

20 $$\eta = \frac{k}{\varepsilon }\sqrt p ,$$

The empirical constants set as default values in model were $\eta_{0} = 4.38$, $\beta_{0} = 1.92$, $c_{1} = 1.44$, $c_{2} = 1.92$, $C_{\mu } = 0.09$, $\sigma_{k} = 1$ and $\sigma_{\varepsilon } = 1$.

#### Cavitation model

Simerics-MP + adopts a cavitation model based on the full cavitation model developed by Singhal et al.^38^, where the working fluid in the cavitating flow is assumed to be a mixture of liquid, vapor, and non-condensable gases (NCGs). The cavitation model describes the cavitation vapor distribution using the following formula:

21 $$\frac{\partial }{\partial t}\int\limits_{\Omega } {\rho f_{v} \cdot d\Omega } + \int\limits_{\sigma } {\rho \left( {\left( {\overrightarrow {\nu } - \overrightarrow {{\nu_{\sigma } }} } \right) \cdot \overrightarrow {n} } \right)f_{v} d\sigma = } \int\limits_{\sigma } {\left( {D_{f} + \frac{{\mu_{t} }}{{\sigma_{t} }}} \right)\left( {\nabla f_{v} \cdot \overrightarrow {n} } \right)d\sigma } + \int\limits_{\Omega } {\left( {R_{e} - R_{c} } \right)d\Omega } ,$$

The vapor generation term $R_{e}$ and the condensation rate term $R_{c}$ are modeled as follows:

22 $$R_{e} = \left\{ {\begin{array}{*{20}l} {C_{e} \rho_{l} \rho_{v} \sqrt {\frac{2}{3}\frac{{\left( {p - p_{v} } \right)}}{{\rho_{l} }}} \left( {1 - f_{v} - f_{g,f} } \right)} \hfill & {p < p_{v} } \hfill \\ 0 \hfill & {p \ge p_{v} } \hfill \\ \end{array} } \right.,$$

23 $$R_{c} = \left\{ {\begin{array}{*{20}l} 0 \hfill & {p < p_{v} } \hfill \\ {C_{c} \rho_{l} \rho_{v} \sqrt {\frac{2}{3}\frac{{\left( {p - p_{v} } \right)}}{{\rho_{l} }}} f_{v} } \hfill & {p \ge p_{v} } \hfill \\ \end{array} } \right.,$$

The constants $\sigma_{t}$, $C_{e}$, and $C_{c}$ were all set to 1 as default.

Based on the model proposed by Singhal et al. Simerics-MP + provides different models for users to select based on different physical assumptions in the computational domain of the NCGs, which makes the cavitation model more robust and the computational results more convergent. The “equilibrium dissolved gas model” was adopted in this paper. It determines the mass fraction of NCGs dissolved in the liquid using the transport of the gases and assuming that the dissolved gases were at equilibrium, which is based on the local pressure and the equilibrium dissolved mass fraction at a dissolved gas reference pressure. The specific equations are as follows:

24 $$\begin{aligned} \frac{\partial }{\partial t}\int\limits_{\Omega } {\rho f_{g,d} \cdot d\Omega } + \int\limits_{\sigma } {\rho \left( {\left( {\overrightarrow {\nu } - \overrightarrow {{\nu_{\sigma } }} } \right) \cdot \overrightarrow {n} } \right)f_{g,d} d\sigma } & = \int\limits_{\sigma } {\left( {D_{g,d} + \frac{{\mu_{t} }}{{\sigma_{g,d} }}} \right)\left( {\nabla f_{g,d} \cdot \overrightarrow {n} } \right)d\sigma } \\ & \quad - \int\limits_{\Omega } {\left( {\frac{{\rho (f_{g,d} - f_{g,d\;equil} )}}{\tau }} \right)d\Omega } + \int\limits_{\Omega } {\left( {S_{g,d} } \right)d\Omega } , \\ \end{aligned}$$

25 $$f_{g,d\;equil} = \frac{p}{{p_{d,equil,ref} }} \cdot f_{d,equil,ref} ,$$

26 $$f_{g,d} + f_{g,f} = const,$$

where $f_{g,d}$ is the mass fraction of the dissolved NCGs, $D_{g,d}$ is diffusivity of the dissolved NCGs, $\sigma_{g,d}$ is the dissolved NCG Schmidt number, $f_{g,d\;equil}$ is the equilibrium mass fraction of the dissolved NCGs at the current cell pressure $p$, $f_{d,equil,ref}$ is the equilibrium mass fraction of the dissolved NCGs at the reference pressure $p_{d,equil,ref}$, and $\tau$ is the time, which approaches zero so that the mass transfer is nearly instantaneous.

### Initial and boundary conditions

The initial and boundary conditions of the model were consistent with that of the subsequent experiments in the following paper, which allows the results to be compared to verify the effectiveness of the simulation. The specific values are shown in Table 3. The fluid medium was No. 46 hydraulic oil. As the bulk modulus of oil is greatly affected by the actual working conditions, the value in the simulation was the corrected value of the bulk modulus of oil from experiments. To accurately represent the oil in the simulations, the Roelands equation was used to determine the dynamic viscosity $\mu$ of No. 46 hydraulic oil at the simulation pressure and temperature^39^:

27 $$\mu { = }0.0457\exp \left\{ {6.58 \times \left[ {\left( {1 + 5.1 \times 10^{ - 9} P} \right)^{{2.3 \times 10^{ - 8} }} \times \left( {\frac{T - 138}{{303 - 138}}} \right)^{ - 1.16} - 1} \right]} \right\}$$

**Table 3:** Initial and boundary conditions of model.

Parameters	Symbol	Value
Density	$\rho$	878 kg/m^3^
Temperature	$T$	313.15 K
Inlet pressure	$P_{in}$	0.2 MPa
Outlet pressure	$P_{out}$	2–12.5 MPa
Pump speed	$n$	800–1800 r/min

### Grid independence verification

Grid independence verification was carried out after the pre-processing. Three groups of gradually refined grid were divided into coarse, normal and fine, the inlet and outlet pressure were set to 1 atm, the speed was set to 1200 r/min, and the average flow rate of the gear pump was monitored in the calculation software. The specific results are shown in Table 4.

**Table 4:** Comparison of average flow rate under different grid elements.

Grid quality	Number of grid elements	Number of nodes	Average flow rate (L/min)
Coarse	142,079	386,965	29.131
Normal	486,020	1,004,034	29.917
Fine	1,623,200	2,189,216	30.152

At present, there are many methods to evaluate numerical error caused by grid quality^40–42^. However, the most widely used method is the Grid Convergence Index (GCI) method proposed by Roache^43^, which indicates an error band on how far the solution is from the asymptotic value and a small value of GCI indicates that the computation is within the asymptotic range. This paper used this method to study the grid convergence. The detailed steps to calculate GCI are as follows:

For three sets of grids from sparse to dense, the results should satisfy the monotone convergence condition, that is $0 < \left( {f_{3} - f_{2} } \right)/\left( {f_{2} - f_{1} } \right) < 1$, then the following formula is satisfied:

28 $$f_{k} = f_{exact} {\text{ + g}}_{p} h_{k}^{p} + O\left( {h_{k}^{p + 1} } \right)\left( {k = 1,2,3} \right)$$

Subscripts $k$($k = 1,2,3$) represent three sets of grids from sparse to dense respectively, $f_{exact}$ is the numerical exact solution, $f_{k}$ is the numerical discrete solution, $h_{k}$ is the grid scale, $p$ is the order of convergence, $g_{p}$ is the $p$- order error coefficient that does not vary with the grid. The relative error between two sets of grids is defined as followed:

29 $$\delta_{{r\left( {k,k + 1} \right)}} = \left| {\frac{{f_{k + 1} - f_{k} }}{{f_{k + 1} }}} \right| = \left| {\frac{{\delta_{k,k + 1} }}{{f_{k + 1} }}} \right|$$

If higher order terms in the Eq. (28) are ignored, $p$ can be calculated by the Eq. (30):

30 $$\frac{{\delta_{12} }}{{r_{12}^{p} - 1}} = \frac{{\delta_{23} r_{23}^{p} }}{{r_{23}^{p} - 1}}$$

$r_{k,k + 1}$ is the grid refinement ratio, which can be calculated by the Eq. (31):

31 $$r_{k,k + 1} = \left( {\frac{{N_{k + 1} }}{{N_{k} }}} \right)^{\frac{1}{D}}$$

where is $N_{k}$ the total number of grid elements and $D$ = 3 is the dimension of the flow domain in this paper.

The Richardson extrapolation are performed to obtain an estimate of the numerical exact solution $\widehat{f}_{exact}$:

32 $$\widehat{f}_{exact} = f_{3} + \frac{{f_{3} - f_{2} }}{{r_{23}^{p} - 1}}$$

The GCI on the grid is defined as:

where $F_{s}$ is a factor of safety and $F_{s}$ = 1.25 is recommended for comparisons over three or more grids^44^.

33 $$GCI_{k,k + 1} = F_{s} \frac{{r_{k,k + 1}^{p} \delta_{{r\left( {k,k + 1} \right)}} }}{{r_{k,k + 1}^{p} - 1}}$$

The data in Table 4 are substituted into the Eqs. (28–33), and the final calculation results are shown in Table 5 below.

**Table 5:** Results of the grid convergence analysis.

Order of convergence $p$	estimate of the numerical exact solution $\widehat{f}_{exact}$	$GCI_{1,2}$	$GCI_{2,3}$
2.9202	30.257 L/min	4.71%	1.41%

It can be seen from the above results that the GCI decreases with the grid encryption. The denser the grid is, the closer the discrete result is to the exact solution, so the discrete error is smaller, and the value of GCI becomes smaller, which indicates that the grid encryption is successful. Generally, the calculation accuracy can be met with the GCI value of less than 5%^43^. In order to obtain more accurate simulation results of the pump, the fine grids were chosen because the GCI for grids 2 and 3 is less than that for grids 1 and 2.

### Results and discussion

According to the analysis in “Theoretical flow ripple characteristics” Section of this paper, the periodic change of gear meshing leads to the change of the internal flow channel structure of the gear pair, and the flow ripple will also undergo periodic changes, which makes the flow field of the oil in the pump also undergo periodic changes. The pump studied in this paper has 10 teeth, therefore, when the gear pump rotates a circle, the flow field of oil will occur 10 times of periodic change, in each cycle, the flow state of different positions will be very different, in order to fully understand the whole cycle flow change process of the pump, it is necessary to analyze the flow field in different positions of the pump. The simulation calculation results at speed of 1200 r/min and outlet pressure of 12 MPa were selected for analysis because it was the rated pump condition in which the system could run stably and maintain high volume efficiency under this condition. The setting of boundary conditions and oil properties were referred to “Initial and boundary conditions” Section. In the calculation process, in order to ensure the accuracy of the results and facilitate the effective analysis of the flow field at different moments, the external gear was set to simulate 5 turns, and 720 calculations were made for each turn, that is, one calculation was made for every 0.5° of gear rotation and the results were saved.

#### Pressure field

**Figure 11:**
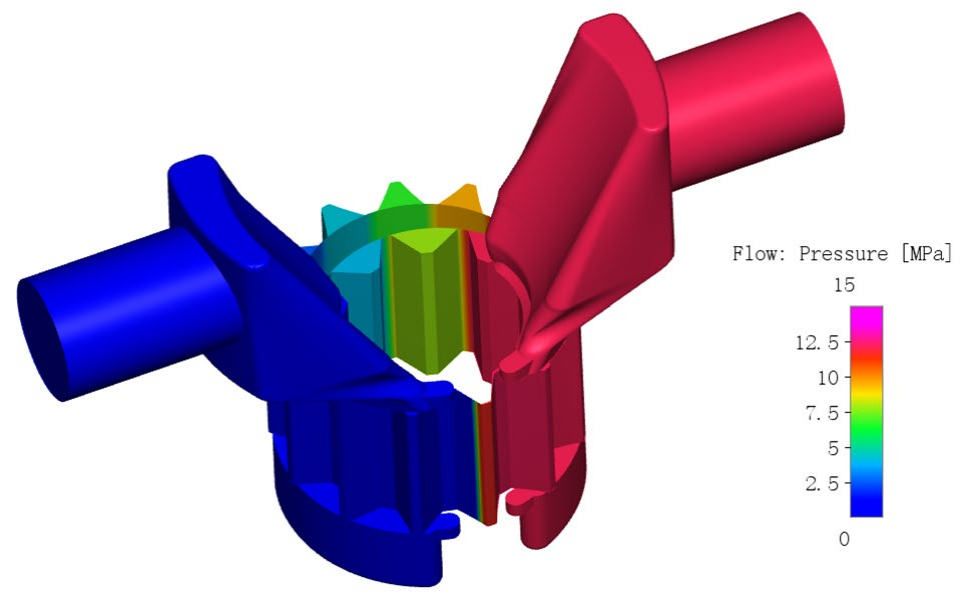
The pressure distribution of the internal flow field of the gear pump.

**Figure 12:**
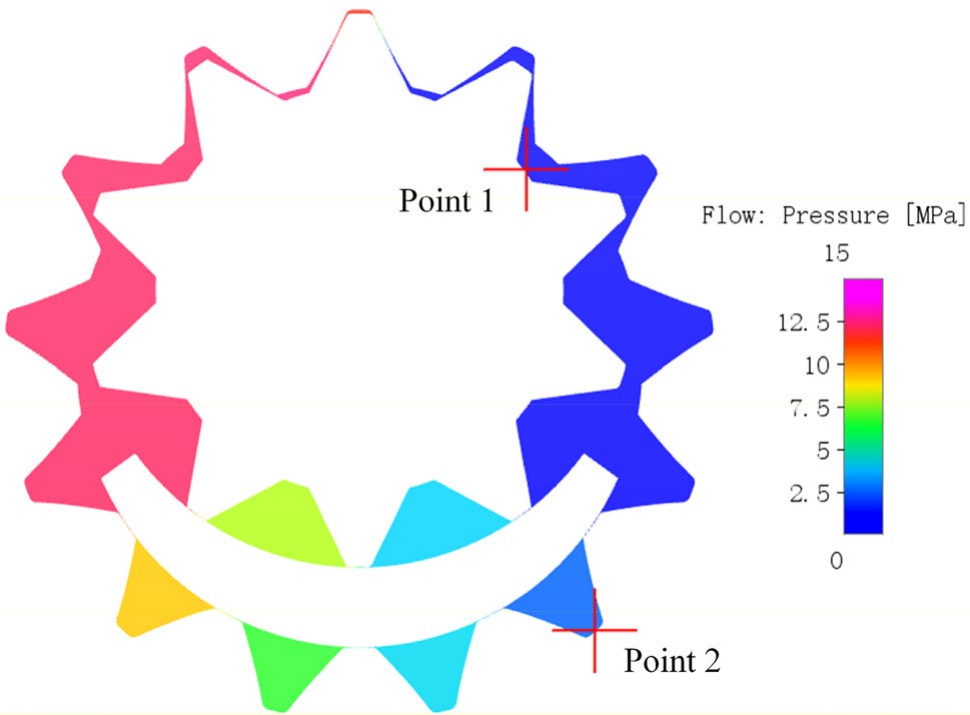
The initial positions of monitoring points.

The pressure distribution of the internal flow field of the gear pump after calculation convergence is shown in Fig. 11, the pressure contours could not represent the change quantitatively due to the pressure gradient of tooth cavity is large, so the monitoring points were inserted into the tooth cavity of the central section of the external gear and the internal gear ring respectively during the simulation for the convenience of analysis, Point 1 in the tooth cavity of the external gear and Point 2 in the tooth cavity of the internal gear ring. The two monitoring cavities are apical cavity and root cavity corresponding to a pair of meshing target teeth, its initial positions are shown in Fig. 12, The external gear is the main driving gear and drives the internal gear ring to move clockwise. The oil suction cavity is on the left and the oil discharge cavity is on the right. When the gear rotates, the monitoring point rotates at the same angular velocity with the corresponding tooth cavity.

**Figure 13:**
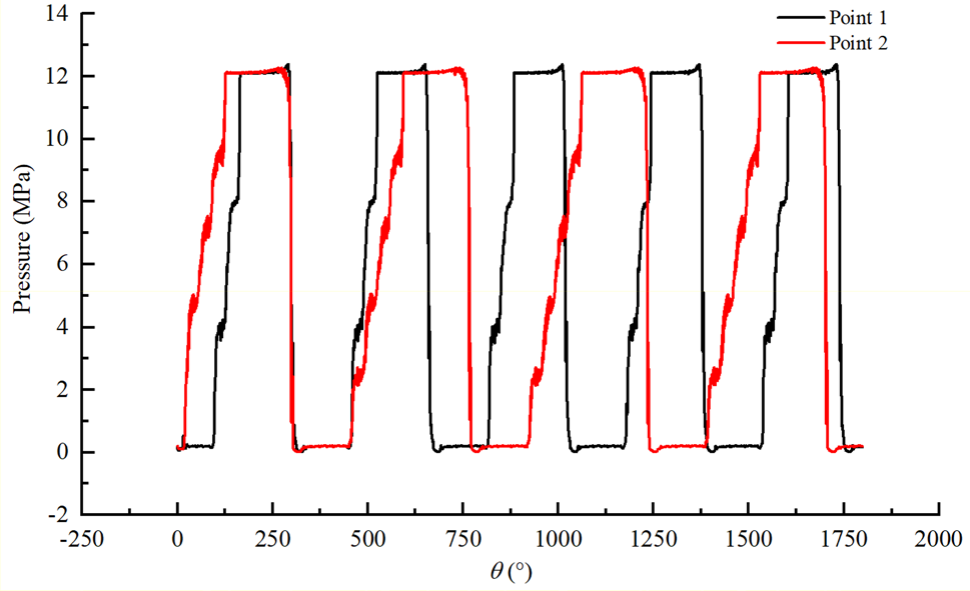
The pressure changes in tooth cavity with rotation angle of external gear.

**Figure 14:**
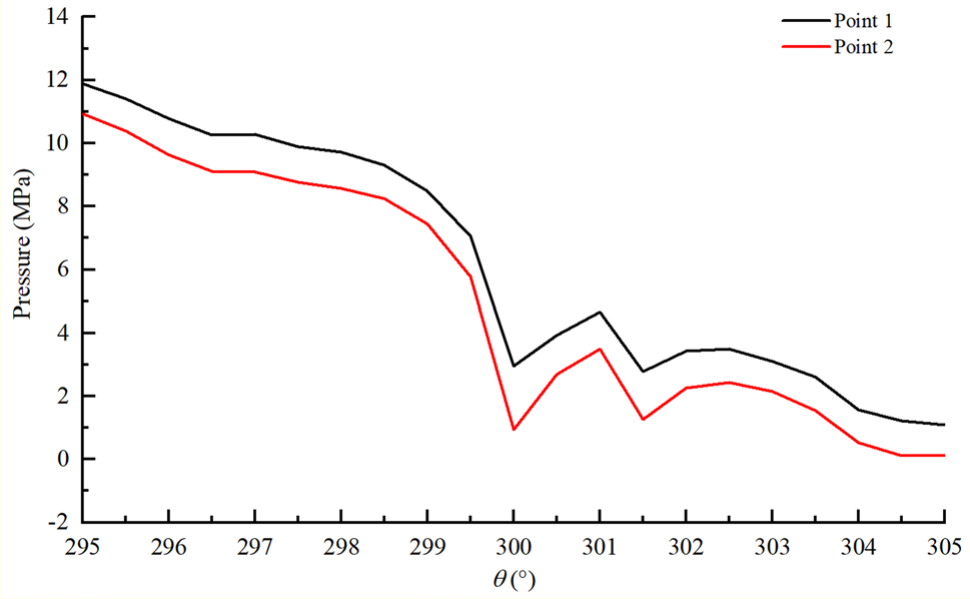
The pressure changes in the tooth cavity in the trapped oil process.

**Figure 15:**
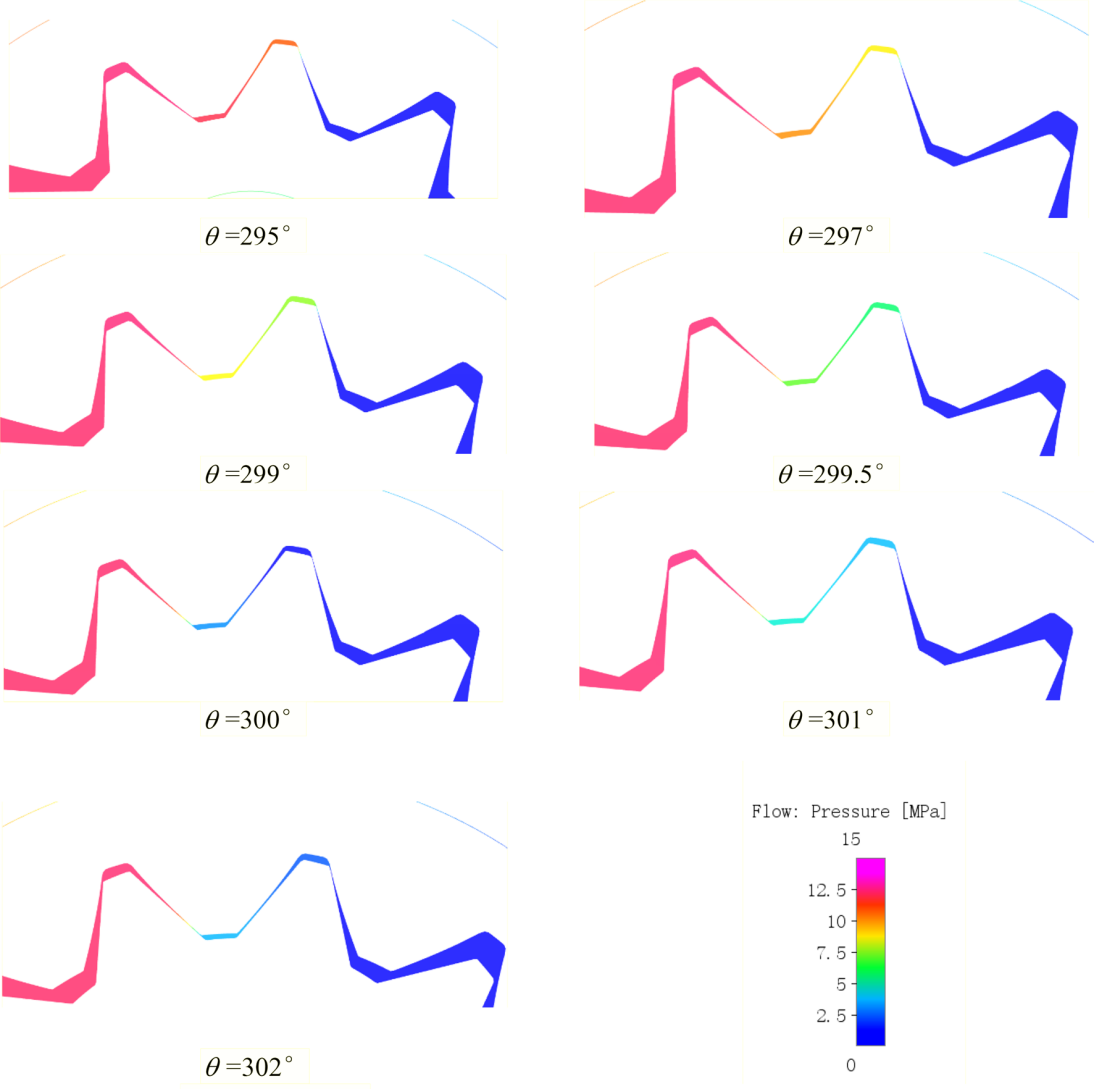
The tooth cavity pressure field in the trapped oil process.

The rotation angle of the external gear is represented by $\theta$. In order to better fit the actual situation, the external gear deflects a small angle to the right, and the internal gear ring is in a symmetrical position about the center line in the initial state of $\theta$ = 0°. When the gear completes 5 turns, the pressure changing curves of tooth cavity with rotation angle is shown in Fig. 13. The following conclusions can be drawn based on Figs. 11 and 13:

1. The pressure changing curves of the external gear and the internal gear ring cavity both showed regular periodic changes. The pressure changing period of the external gear cavity $T_{1}$ = 360°. Since the transmission ratio of the external gear and the internal gear ring $i_{12}$ = 13/10, the pressure changing period of the internal gear ring cavity $T_{2}$ = $T_{1} \times i_{12}$ = 468°.
2. In a single pressure change cycle, when the rotation angle is not more than 90°, the monitoring cavity of external gear is in the oil suction cavity;
  when rotation angle is between 90° and 162°, the monitoring cavity rotated in the transition cavity and began to build pressure, because there is a fixed oil film gap between the crescent plate and the gear pair, the oil leakage causes a certain pressure loss between the adjacent tooth cavities, so the pressure in the tooth cavity gradually increases. The closer the monitored tooth cavity is to the oil discharge cavity, the smaller the pressure loss caused by leakage is and the greater the pressure is.
  When the rotation angle is between 162° and 295°, the monitoring cavity is in the oil discharge cavity; When the rotation angle is between 295° and 302°, the monitoring cavity is in the trapped oil cavity formed by the engagement of the front and back teeth. The difference of tooth shape results in the change of trapped oil pressure of the pump is different from that of the involute gear pump. Therefore, the change of trapped oil pressure of the meshing tooth was discussed and analyzed in detail at point (4) of the conclusion. After the rotation Angle exceeds 302°, the monitoring tooth cavity is connected with the oil suction cavity and enters the oil suction cavity again. After that, the monitoring tooth cavity continues to rotate and return to the starting position for the next cycle. The pressure of the monitoring tooth cavity in the pump suction and oil discharge cavity fluctuates little, and is basically consistent with the pressure of the inlet and outlet. The pressure of the oil suction cavity is about 0.1 MPa, and the pressure of the oil discharge cavity is about 12.1 MPa.
3. In a single pressure change period, the pressure changing trend of the tooth cavity monitored by the internal gear ring is similar to that of the external gear, but the difference is that its pressure change period is longer than that of the external gear. Meanwhile, the number of tooth cavities corresponding to the outer part of the crescent plate is 4, and the number of tooth cavities corresponding to the inner part is 2, so that in the pressure rise stage of the transition cavity, the pressure rise of internal gear ring presents 4 steps while that of the external gear only presents 2 steps.
4. When the rotation angle changes between 295° and 300°, the front and back teeth engage at the same time to form oil trapped phenomenon, which results in a wide range of pressure changes in the tooth cavity. The pressure changes in the tooth cavity during this period are plotted separately, as shown in Fig. 14. The tooth cavity pressure field along with the change of rotation angle when oil trapped phenomenon occurs is shown in Fig. 15, the selected section is the section of the central position of the internal flow field. When the rotation angle is about 295°, the gear pair is about to enter the two-tooth meshing state, and the high-pressure oil enters the meshing line of the front and back teeth and the end cover of the pump to form a closed space, resulting in oil trapped.

It can be seen from Figs. 14 and 15 that the pressure of trapped oil cavity drops sharply and reaches the lowest when the rotation angle is about 300°. Different from involute gear pumps, the pressure of trapped oil cavity pressure does not have a sharp rise stage, because the pump's special tooth shape determines that its trapped oil volume has been increasing in the trapped oil process, only the expansion without compression process, so as to avoid gear, shaft and bearing by a large radial force, improve the stability of the pump operation^10^.

When the rotation angle reaches about 300°, the oil trapped phenomenon ends and the monitoring gear teeth gradually detach. Due to the small clearance on the tooth side at the early stage of the detach process, the high-speed flowing oil generated by the gear rotation cannot flow out smoothly, resulting in a small increase in the pressure of the monitoring tooth cavity, as shown in Figs. 14 and 15. As the gear rotates, the clearance on the tooth side increases gradually, and the pressure in the monitoring cavity falls back to about 0.1 MPa.

#### Velocity field

**Figure 16:**
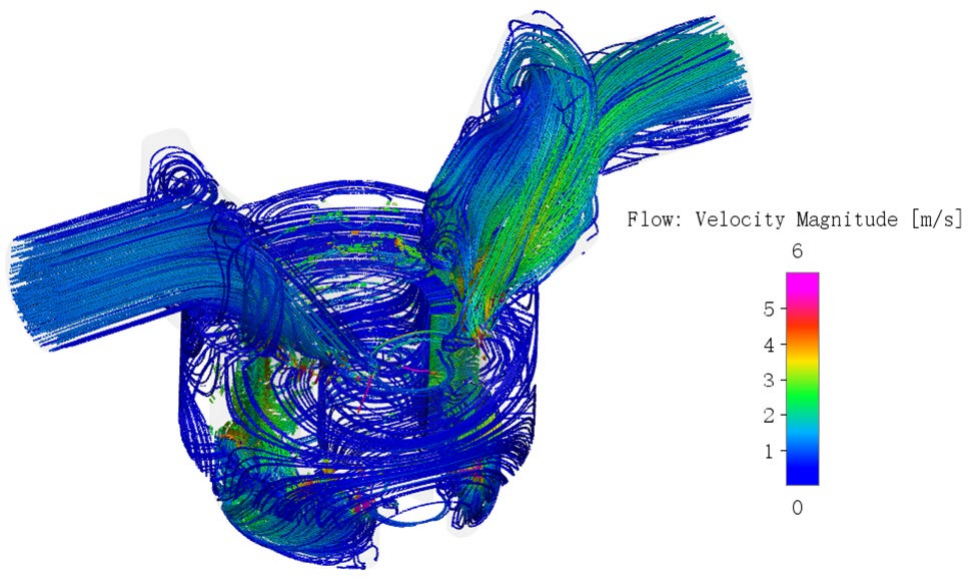
Velocity streamline diagram of the pump.

**Figure 17:**
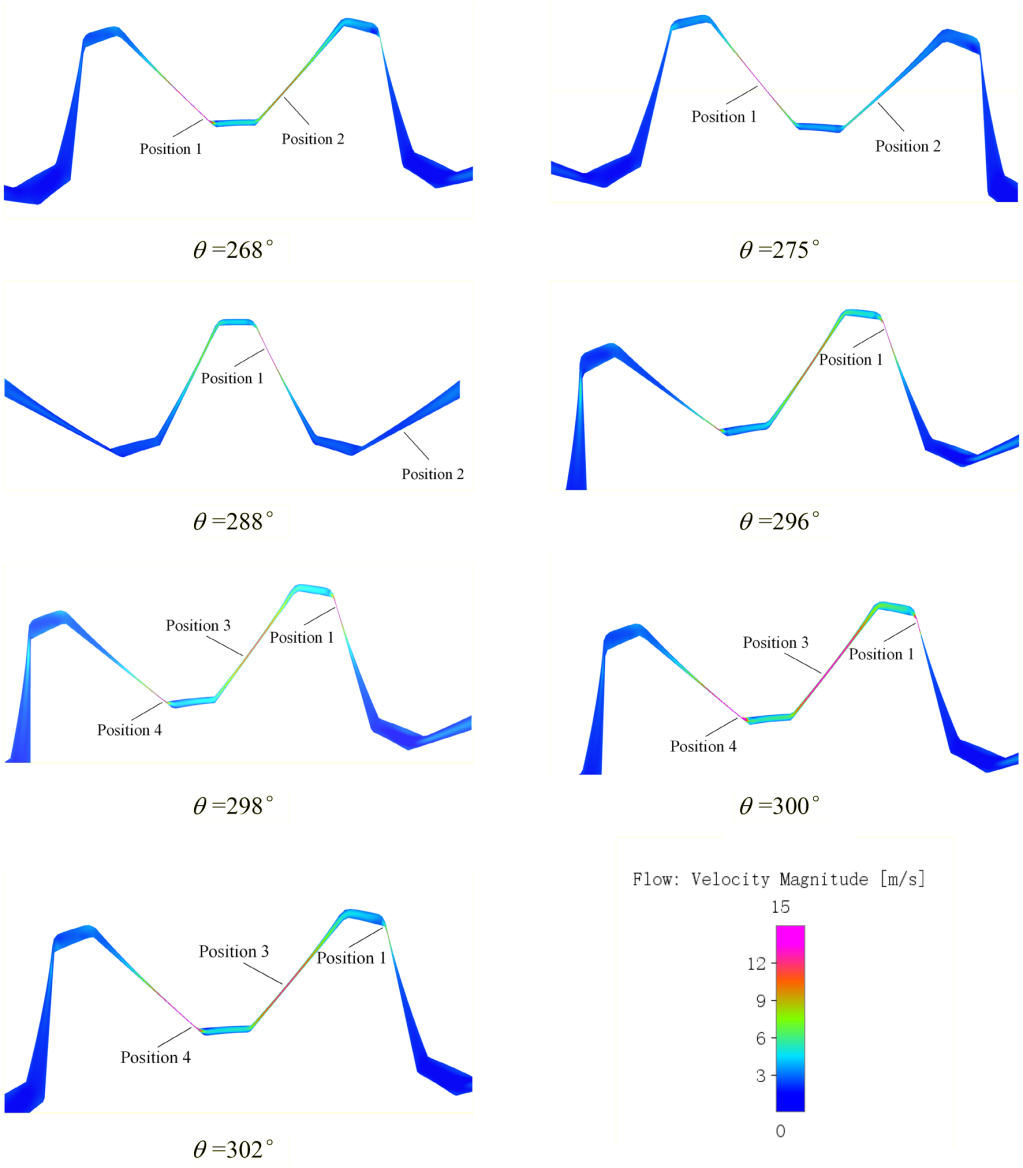
Velocity field in a meshing cycle of the monitoring gear.

The velocity streamline diagram of the pump is shown in Fig. 16, which shows an overview of the velocity distribution and the flow state of the pump. The oil velocity in the rotor area and delivery passageway was significantly higher than that in the oil distribution area and the suction due to the continuous engagement of the gear pair to draw in and discharge oil. As can be seen from the previous analysis, the pressure difference of gear tooth meshing region is large and the meshing gap is very small. The constant change of meshing position will make the speed field in this region constantly change. Therefore, the speed field of the gear tooth meshing region is analyzed emphatically. According to the working principle, the number of external gears of the pump is 10, and 10 periodic changes will occur when the external gears rotate once a week. The corresponding rotation angle range of one meshing cycle is 36°, for the convenience of analysis, the monitoring tooth is the same as that in the pressure field analysis, and the velocity field in one meshing cycle is drawn, as shown in Fig. 17.

When the rotation angle is about 268°, the gear just enters the single tooth meshing state. At this point, the meshing point is located near Position 1, and the oil velocity in this region is the largest, up to 22 m/s. At the same time, the side clearance between the gear in front of the oil suction chamber is small, about 100 um, and the oil extrusion leads to a high oil velocity at Position 2, up to 12 m/s at the center;

When the rotation angle changes between 268° and 296°, the gear is still in the state of single tooth engagement, and the meshing point moves from the tooth root to the tooth crown along the tooth profile of the external gear. As the meshing clearance changes around 15 um with little change, the central flow velocity at the meshing Position 1 is always up to about 20 m/s, but with the further increase of the rotation angle, the teeth are separated and the clearance on the tooth side increases, the oil velocity in Position 2 gradually decreases to 2 m/s, and the oil velocity there is basically the same as that in other tooth cavities of the oil suction cavity.

When the rotation angle changes between 296° and 300°, The gear and a pair of teeth behind it are in a two-tooth meshing state, forming a trapped oil cavity. The extrusion of high-pressure oil in the cavity makes the oil velocity at the central tooth clearance in the cavity as high as 15 m/s at Position 3, As can be seen from the above pressure field analysis, during the process of oil trapped, the volume of cavity content increases, the pressure gradient is large and pressure keeps decreasing, forming a vacuum, and oil needs to be supplemented from the high-pressure oil discharge chamber. Therefore, the oil velocity at the center of the last pair of gear teeth meshing point is as high as 35 m/s at Position 4, the right side of the target gear teeth meshing point is the low-pressure oil suction cavity, and the central velocity is still about 20 m/s with little change at Position 1.

When the rotation angle reaches about 300°, the trapped oil ends and the target gear teeth gradually detach. Due to the small clearance on the tooth side in the early stage of detach process, the oil velocity at Position 1 gradually decreases until the rotation angle reaches 302°, which verifies why the tooth cavity pressure rises slightly at this stage in pressure field.

#### Cavitation

When the liquid is dissolved into the gas, the pressure in the liquid is reduced to the gas separation pressure under the local thermodynamic state, the gas dissolved in the liquid will be precipitated; when the pressure inside the liquid down to the saturated vapor pressure under the local thermodynamic state, the fluid medium will change from liquid phase to gas phase, both of which are called cavitation. In practical engineering, the oil will inevitably dissolve into a certain amount of gas, and the internal pressure of the gear pump changes violently during operation, and its cavitation state is often the coexistence of gas cavitation and vapor cavitation^45^.

**Figure 18:**
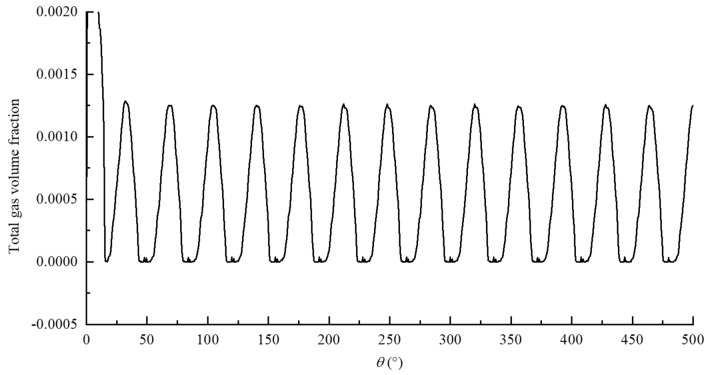
The variation of total gas volume fraction in rotor region with rotation angle.

**Figure 19:**
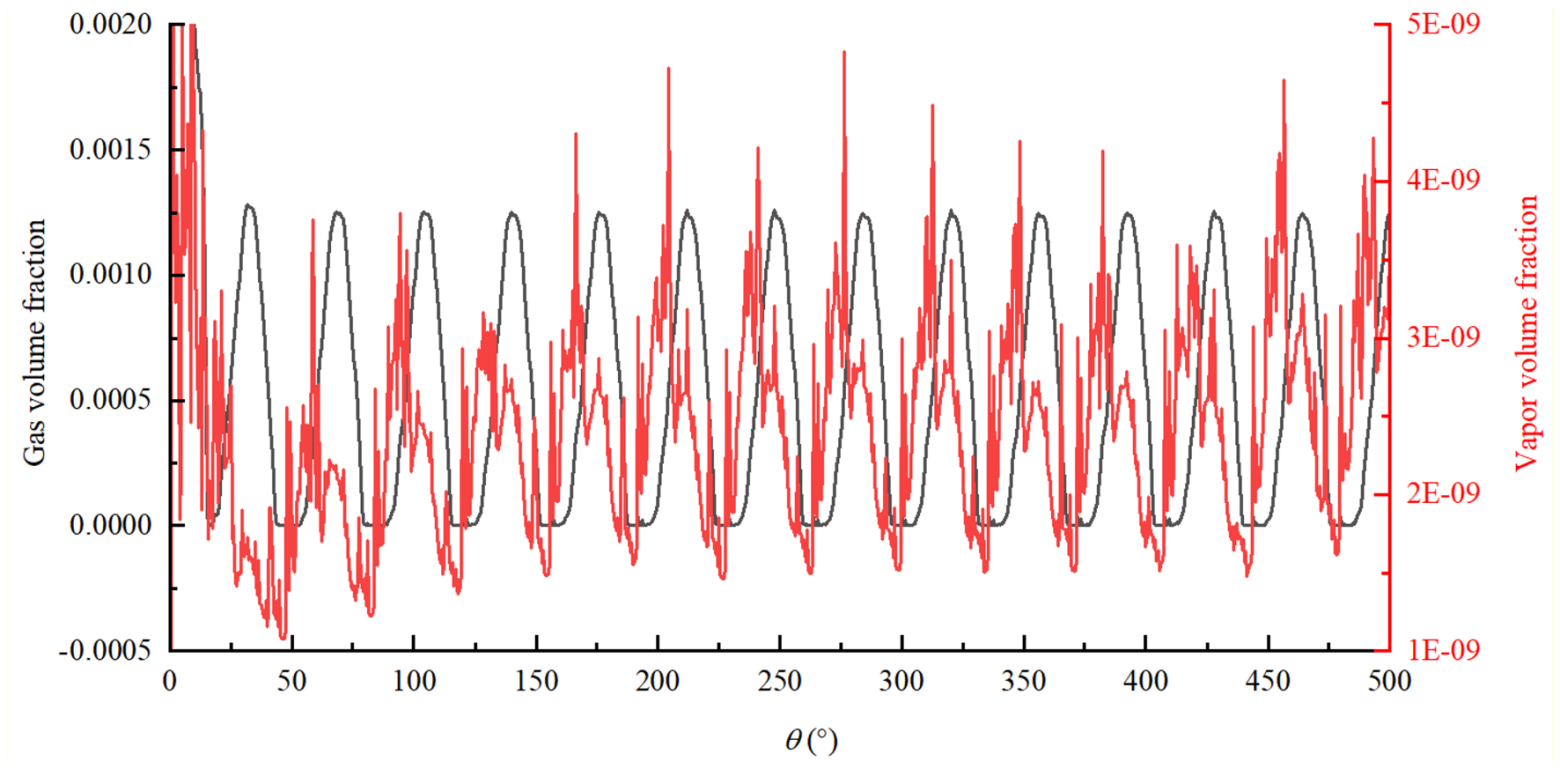
The variation of gas volume fraction and vapor volume fraction in rotor region with rotation angle.

**Figure 20:**
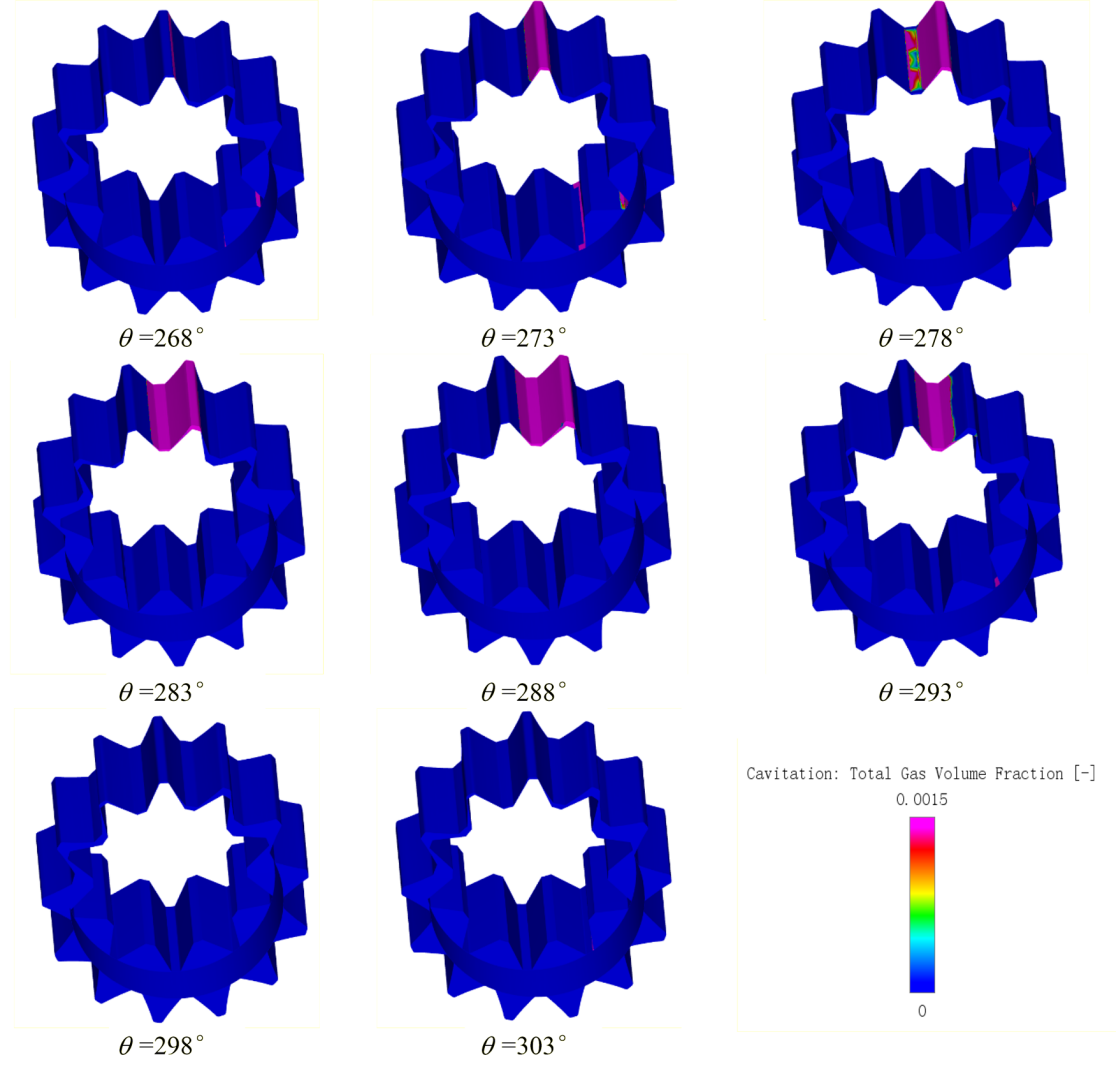
The cavitation fields of total gas volume fraction during a mesh cycle.

In the model used in this paper, gas cavitation was mainly caused by the dissolved NCGs into the oil, when the local pressure was lower than the reference pressure, the dissolved NCGs precipitated into the free NCGs, and vapor cavitation was mainly caused by the fact that the local pressure was lower than the liquid vapor pressure, that part of oil is converted into oil vapor. Gas cavitation was related to the gas volume fraction, and vapor cavitation was related to the vapor volume fraction in the fluid domain. Simerics-MP + accounted for the changes in the gas volume fraction caused by both forms of cavitation and the total gas volume fraction $f_{t}$. In the cavitation model selected in this paper, $f_{t}$ was the sum of the gas and vapor volume fractions, which reflected the ratio of the volume of all the gases to the total fluid volume, that is, the overall degree of cavitation in the internal flow field. The gas separation pressure $p_{d,equil,ref}$ of oil is 1 atm, the saturated vapor pressure $p_{v}$ is 400 Pa, and the gas mass fraction $f_{g,d}$ in the oil is $9 \times 10^{ - 5}$, which are set by default. As can be seen from the previous analysis, the rotor region is changes with the large pressure and velocity gradient, so the study focuses on the cavitation characteristics in the rotor region. Figure 18 shows the variation of total gas volume fraction in the rotor region with rotation angle. In order to further explore the cavitation evolution law during operation, the variation of gas volume fraction and vapor volume fraction were drawn separately, and the cavitation fields of total gas volume fraction during a mesh cycle were also drawn, as shown in Figs. 19 and 20 respectively. for the convenience of analysis, the monitoring tooth is the same as that in the pressure and velocity field.

Based on Figs. 18, 19 and 20, the following conclusions can be drawn about cavitation characteristics of the pump:

1. As can be seen from Fig. 18, the total gas volume fraction in rotor region changes periodically with the rotation of the external gear. The volume fraction fluctuates within the range of 0–0.13% with a change period of about 36°, which is just the angle required to rotate a pair of meshing teeth;
2. As can be seen from Fig. 19, the gas volume fraction released by gas cavitation is almost the same as that of the total gas in the process of cavitation, while the vapor volume fraction is almost 0, indicating that gas cavitation plays a dominant role in the pump when cavitation occurs, and the gas generated by vapor cavitation is very little, almost none. Also, it indicates that there is no pressure drop below 400 Pa in the rotor region under this working condition.
3. Combined with Figs. 18 and 20, it can be seen that cavitation gas is generated at almost all angles in the rotation process, but there are certain differences in the amount of gas generated at different angles, the location and range of the region where the gas is generated. The location of cavitation is concentrated in the region close to the meshing point of the oil suction cavity, and the emergence of cavitation phenomenon at each position will undergo an evolution process of initiation, development and intensification, and gradual dissipation. During this evolution process, the cavitation intensity (the amount of cavitation gas generated) will undergo a weak-strong–weak process. The cavitation range (the area where cavitation gas is produced) will undergo a process from small to large to small, and the corresponding degree of cavitation will also undergo a process from mild cavitation to severe cavitation to mild cavitation. Combined with the working principle, pressure and velocity analysis results of the pump, a pair of gear teeth in front of the meshing point are detached, the volume expansion of the oil suction cavity at the place causes the oil suction empty, which reduces the pressure of the oil suction cavity, on the other hand, after the oil passes through the narrow gap between the teeth, the oil velocity is too large, which leads to the further reduction of local pressure. When the pressure is lower than the gas separation pressure, gas cavitation will occur.

**Figure 21:**
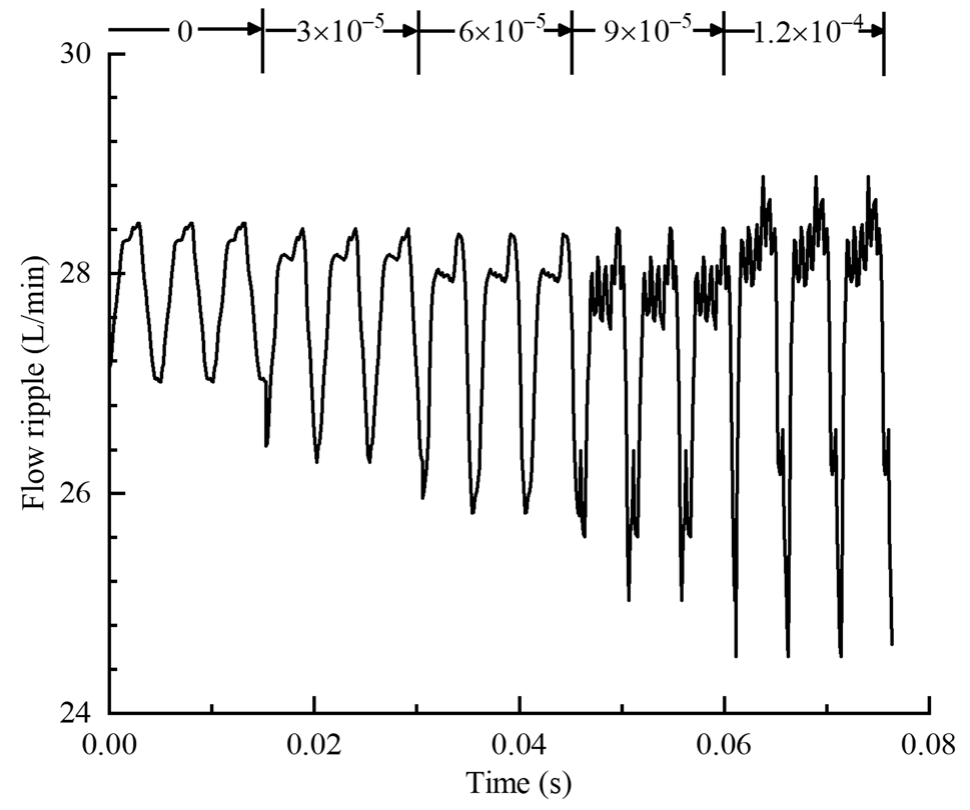
Flow ripple results under different gas volume fraction.

**Figure 22:**
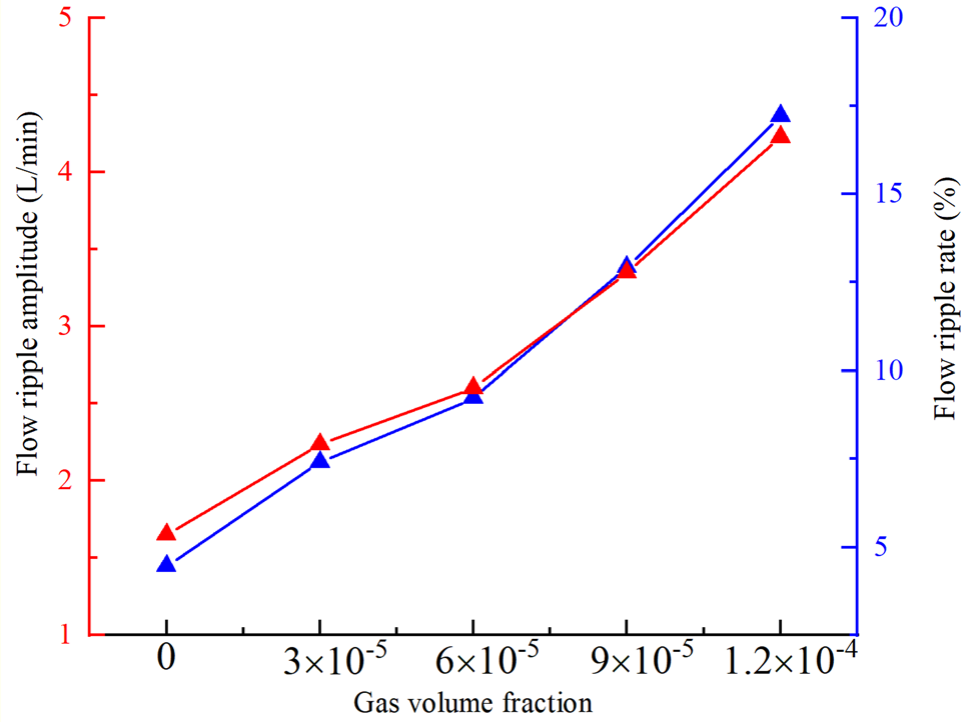
Influence of gas volume fraction on flow ripple characteristics.

According to the previous analysis, when the gas in the oil is separated out, the fixed volume in the oil suction chamber is occupied by bubbles, resulting in a decrease in the effective volume in the chamber, and the amount of bubbles produced is different with the degree of cavitation of the pump, which will certainly affect the pump outlet oil flow ripple characteristics. In order to control the cavitation degree and ensure that the regulating parameters will not affect the other performance of the pump, it can only be achieved by controlling the gas mass fraction in the oil. Multiple groups of different gas mass fraction were set and numerically calculated. The corresponding cavitation degree was from weak to strong, and the settings of other boundary conditions remained unchanged and consistent. The flow ripple results and the flow ripple characteristics at the pump outlet under different gas mass fraction were shown in Figs. 21 and 22.

With the increase of gas mass fraction, the flow ripple amplitude at the pump outlet increases, the average flow rate of output decreases, and the flow ripple rate increases. As the degree of cavitation intensifies, more bubbles are precipitated in the oil, when these bubbles are transported to the high-pressure part of the rotor region, the bubbles will compress and break under the action of high pressure, which reduces the stability of the oil flow and increases the flow ripple amplitude. At the same time, the bubbles generated by cavitation will occupy the volume of the suction and discharge chamber, so that the effective working volume of the pump decreases, when the cavitation intensifies, the gas generated will increase, and the volume of the cavity occupied will increase, leading to the decrease of the average flow rate of output and the increase of the flow ripple rate.

**Figure 23:**
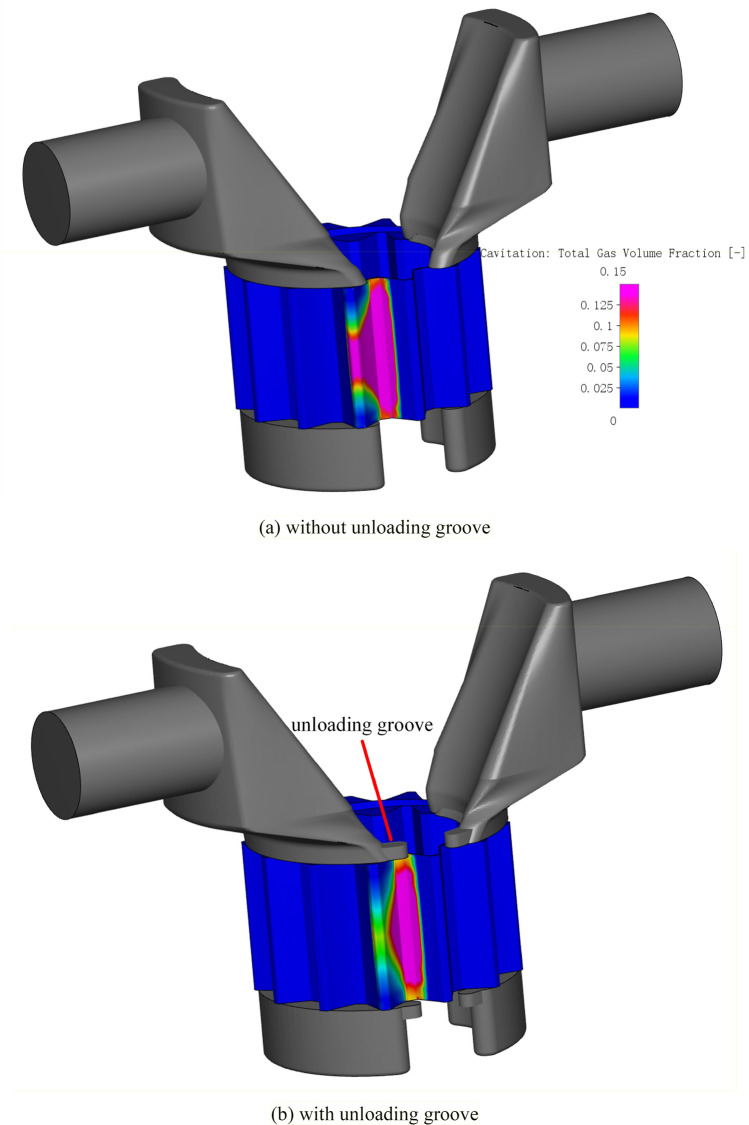
Cavitation field (**a**) without unloading groove; (**b**) with unloading groove.

**Figure 24:**
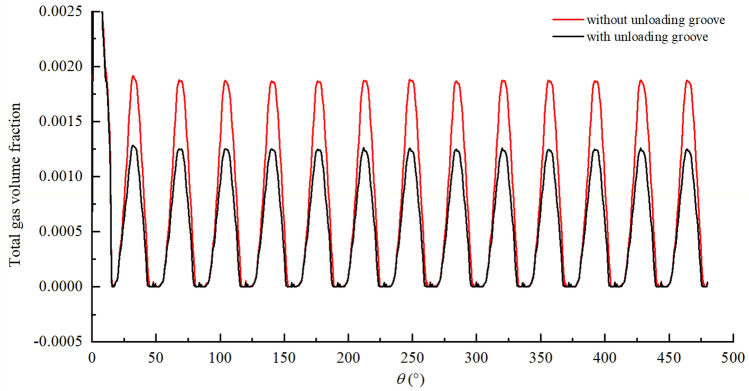
The variation of gas volume fraction in the rotor region with and without unloading groove.

Therefore, suppressing cavitation can reduce flow ripple at the pump outlet and improve volumetric efficiency. In general, cavitation is alleviated by setting unloading groove at locations where cavitation is severe, as shown in Fig. 23, on the one hand, when vacuum-suction occurs, the unloading groove can timely supplement the oil from the oil suction cavity through the end face of the gear to the tooth cavity, on the other hand, the existence of the groove can alleviate the oil speed and improve the stability of oil suction and discharge. The effect of the unloading groove on the cavitation of the pump is explained by comparing the cavitation of the pump under the two conditions with or without the unloading groove, Fig. 23 shows the cavitation field without and with the unloading groove. By comparison, it can be seen that the cavitation position of the pump is basically the same under the two conditions, but the cavitation degree of the area near the unloading groove is obviously weakened with the unloading groove. Figure 24 shows the variation of gas volume fraction in the rotor region without and with unloading groove. The gas volume fraction fluctuates between 0 and 0.19% in gear pump without unloading groove, but fluctuates between 0 and 0.13% in gear pump with unloading groove. The gas volume fraction in the rotor region with unloading groove is significantly lower than that without unloading groove at the same rotation angle. It can be seen that reasonable setting of unloading groove can effectively reduce the cavitation degree of the pump.

## Experimental verification

### Experimental test method and apparatus

The secondary source method was adopted as an international standard to experimental measurement of the flow ripple of hydraulic pumps with high accuracy^26^, so it was used to obtain results experimentally to provide a basis for comparison, thus validating the simulation model.

**Figure 25:**
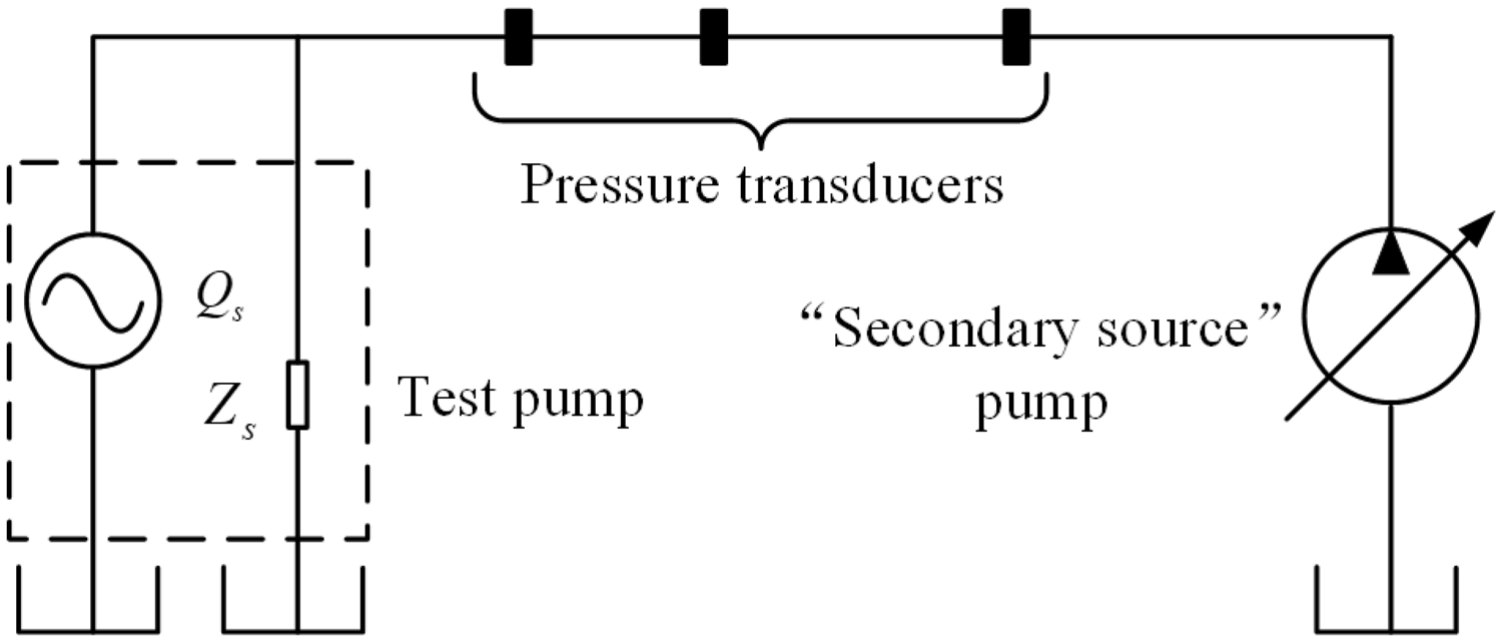
Schematic illustrating the secondary source test method.

The schematic of the secondary source test setup is shown in Fig. 25. The test pump acts as a pump source flow ripple $Q_{S}$ in parallel with the pump source impedance $Z_{S}$. And the flow ripple is assumed to generate at the exit of the pump^3^. Because the characteristics of the source impedance are unknown, a “secondary source” pump with known frequency characteristics is introduced to determine the source impedance, and the pressure ripple at different positions of the connected pipes between the test pump and secondary source pump are synchronously collected to deduce the source flow ripple.

**Figure 26:**
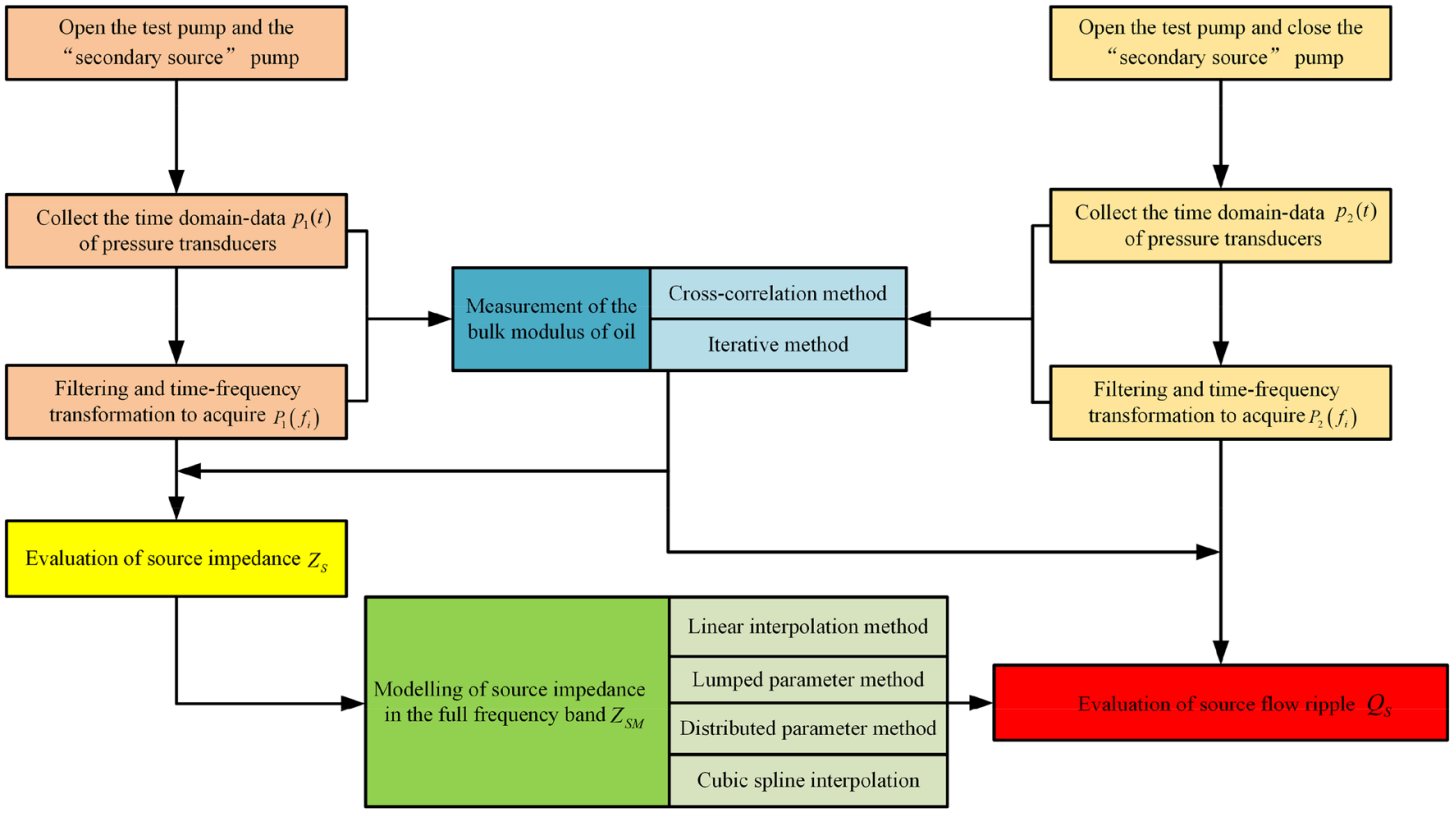
Testing process and data processing flow chart of the secondary source method.

The test process and data processing flow chart of the secondary source method are shown in Fig. 26. During the test, relevant precautions should be strictly followed according to ISO standard, also, the transducer positions and the detailed procedure for calculating the impedance and flow ripple should comply with standard to prevent the failure of the test data. In general, the secondary source method testing process is divided into the following three steps:

1. Evaluation of source impedance $Z_{S}$. The test pump and the secondary source pump are opened. The time-domain data from the pressure transducers is collected, and the source impedance of the test pump at the harmonic frequency $f_{i}$ of the secondary source pump is then computed.
2. Modelling of source impedance in full frequency band $Z_{SM}$. Different fitting methods^4,26^ should be further used to obtain the pump source impedance curve of the test pump in the full frequency band.
3. Evaluation of source flow ripple $Q_{S}$. The test pump was opened, the secondary source pump was closed, and the time-domain data of the pressure transducers was collected. The source flow ripple of the test pump under this working condition was then determined.

**Figure 27:**
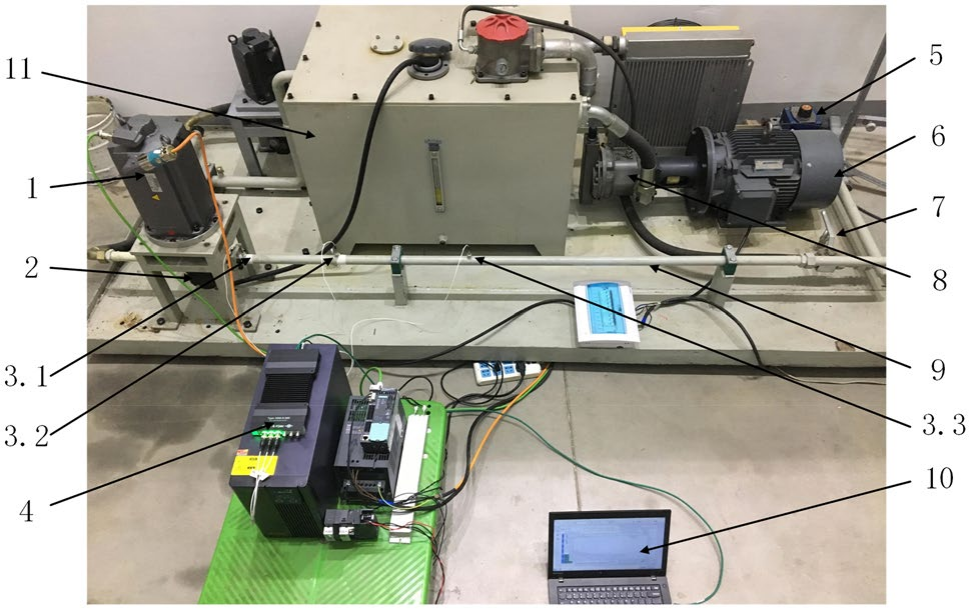
Test platform of the secondary source method: 1—Servo motor; 2—Test pump; 3.1—Pressure transducer No. 1; 3.2—Pressure transducer No. 2; 3.3—Pressure transducer No. 3; 4—Data acquisition module; 5—Variable proportional throttle valve; 6—Three-phase asynchronous motor; 7—Stop valve; 8—Piston pump; 9—Test line; 10—Upper computer; 11—Tank.

The components selected for the test platform are summarized in Table 6, and the test platform is shown in Fig. 27. To achieve the functions described above, a hard, long, straight pipe was installed at the outlet of the test pump, and three high-frequency dynamic pressure transducers were installed on the pipe to directly measure the pressure ripples. A piston pump was set as a secondary source pump in the loading part of the hydraulic system to measure the pump source impedance of the test pump. To measure the effective bulk modulus of the oil, the iterative method or cross-correlation method^46^ can be used. The iterative method recommended by the ISO standard was used in this study. The system was designed to measure and adjust the working parameters. This design included a proportional throttle valve to adjust the working pressure of the pump, a servo motor to control and adjust the speed of the pump, and flow, pressure, and temperature transducers to monitor and measure the system parameters.

**Table 6:** Components selected for test platform.

Components	Type	Basic performance parameters
Secondary source pump	Piston pump	Nine plungers, displacement: 71 ml/r
Motor of secondary source pump	Three-phase asynchronous motor	Rated speed: 1475 r/min
Motor of test pump	Servo motor	Rated speed: 1500 r/min, torque: 50 N m
Pressure transducer	PCB 113B26	Resonance frequency: 500 kHz, range: 0.014 kPa–3.45 MPa, sensitivity: 1.45 mV/kPa
Test line	Rigid, long, straight pipe	Inner diameter 25 mm, wall thickness 1.5 mm
Data acquisition module	BK 3050-A-060	Sampling frequency: 51.2 kHz

From the above it could be known that the uncertainty of this test mainly comes from the following aspects: (1) The inevitable fluctuation of test parameters of the test platform, such as temperature, density, flow, pressure, etc.; (2) The measurement performance of the instrument, such as sensor sensitivity, data acquisition system sampling rate and resolution, etc. (3) Approximations and assumptions in measurement methods and procedures: such as estimation algorithms for calculating pump source impedance and flow ripple, etc.

### Test data processing and analysis

**Figure 28:**
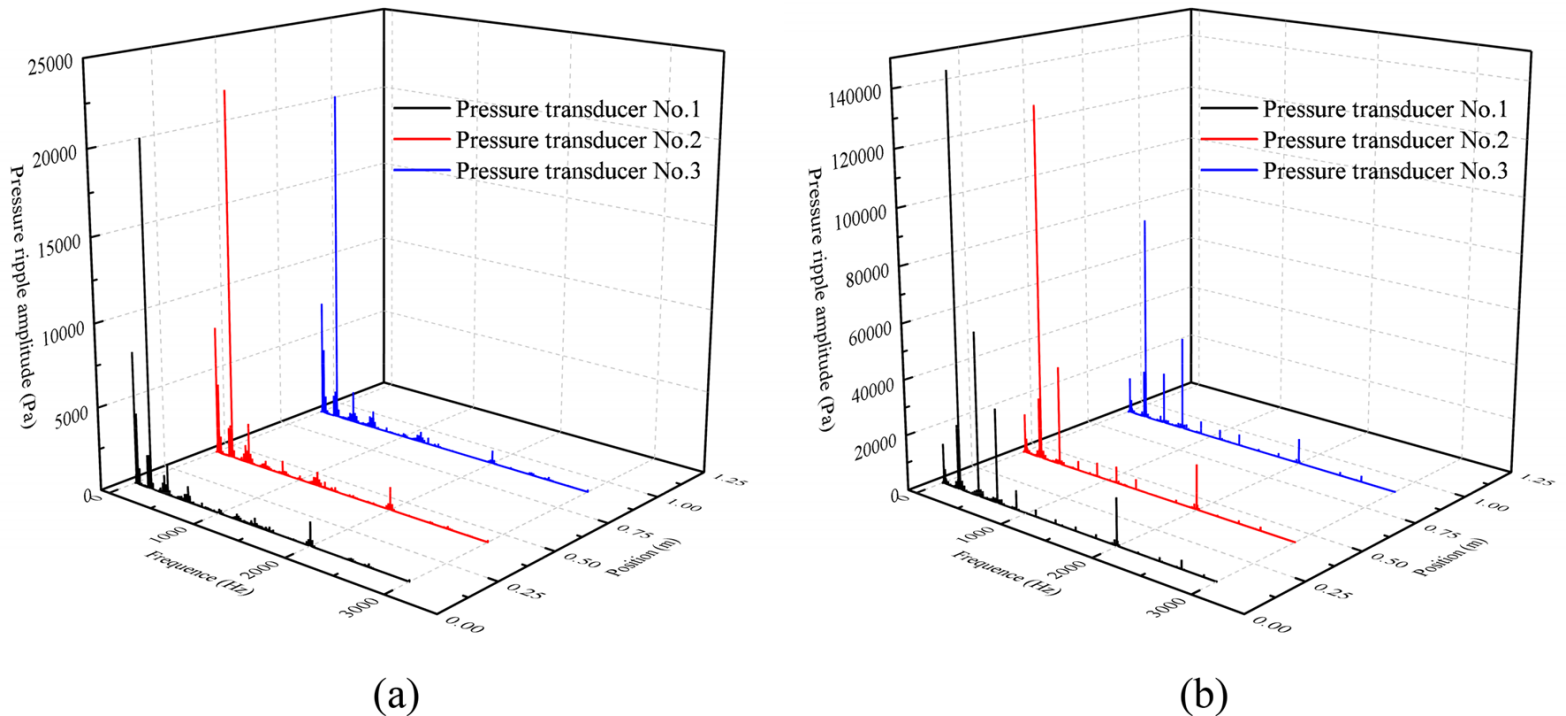
(**a**) $$P_{1} \left( f \right)$$, and (**b**) $$P_{2} \left( f \right)$$.

The experimental parameters were consistent with the simulation boundary conditions. The hydraulic system used No. 46 hydraulic oil, and the oil temperature was controlled to about 313.15 K in the test. The positions of the three pressure transducers were $x_{1} = 0.1$ m, $x_{2} = 0.43$ m, $x_{3} = 0.9$ m in sequence. The range of pump speed $n$ was 800–1800 r/min, and the range of outlet pressures $P_{0}$ was 2–12.5 MPa. With the steady operation of the test pump at 1200 r/min and the outlet pressure at 5 MPa as an example, the detailed data processing steps are discussed below. $P_{1} \left( f \right)$ and $P_{2} \left( f \right)$ could be obtained after filtering and time–frequency transformation of the original time-domain data $p_{1} \left( t \right)$ and $p_{2} \left( t \right)$, which are shown in Fig. 28.

**Figure 29:**
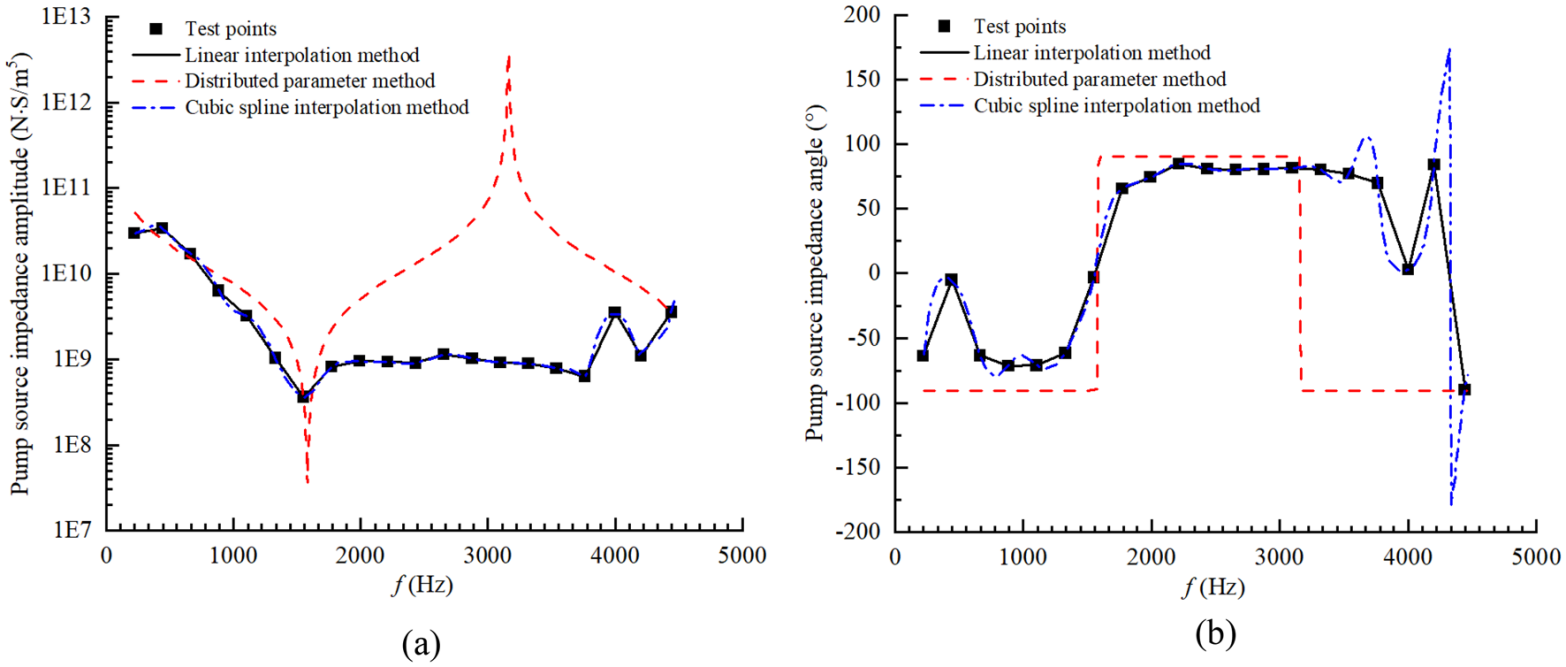
(**a**) Amplitude–frequency curve of $Z_{SM}$ and (**b**) phase-frequency curve of $Z_{SM}$.

The pressure ripple $P_{1} \left( {f_{1,i} } \right)$ of the secondary source pump's harmonic frequency $f_{1,i}$ was extracted to calculate the effective bulk modulus of the oil and the test pump source impedance $Z_{S,i}$, then different curve fitting methods were used to obtain an estimate of the full-band impedance of the pump source $Z_{SM}$. As shown in Fig. 29, the linear interpolation method could reflect the variation trends of the pump source impedance in the whole frequency band, but the fitted curve was not smooth enough, and the error was large for some frequency bands. The distributed parameter method could predict the anti-resonance characteristics of the test pump^4^, but in the high frequency band, the pump source impedance fitted curve diverged, resulting in a large error. Thus, it was not suitable for the test pump. The cubic spline interpolation method could not only reflect the trend of the pump source impedance but could also makes full use of the information contained in the test data. This approach achieved good smoothness of the fitted curve and a high estimation accuracy, so it was adopted in this study.

**Figure 30:**
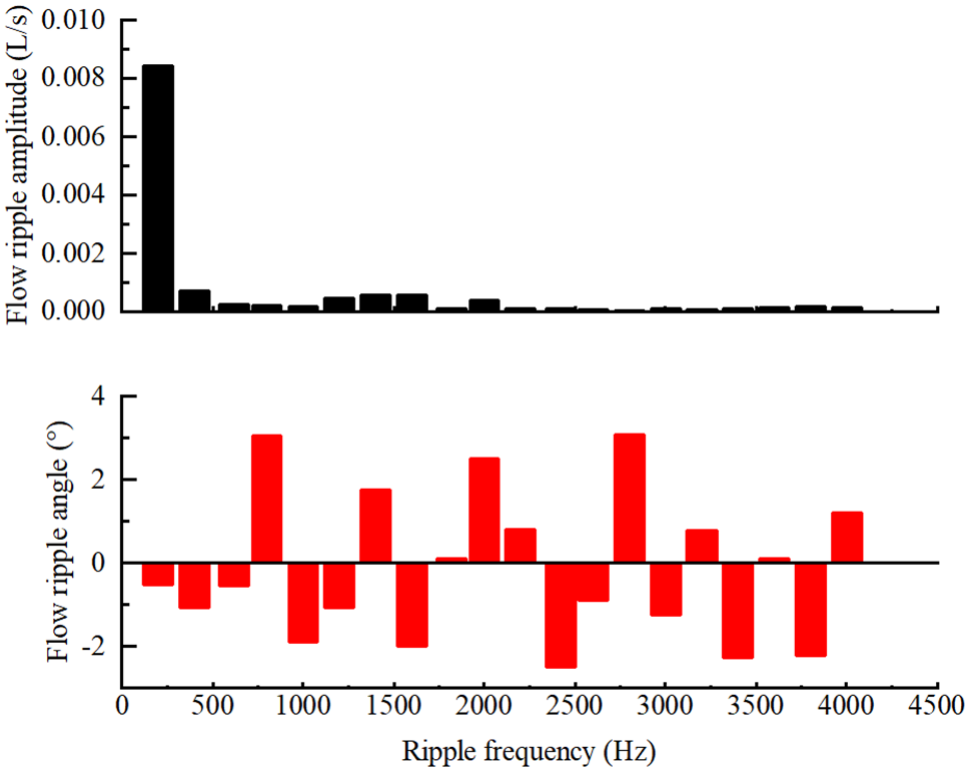
Flow ripple amplitude and phase at the first 20 orders of the ripple frequency.

**Figure 31:**
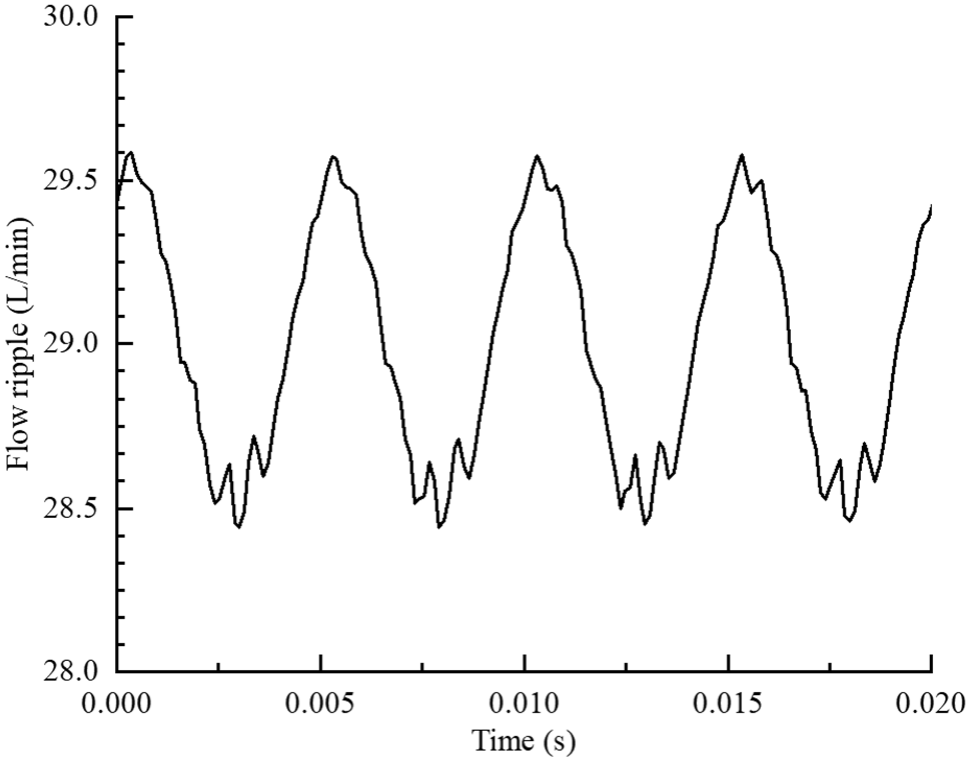
Time-domain curve of flow ripple ($n$ = 1200 r/min, $P_{0}$ = 5 MPa).

The test pump source impedance $Z_{SM,i}$ at the test pump ripple frequency $f_{2,i}$ was extracted by using the estimated full-band impedance $Z_{SM}$ curve of the working condition. The effective bulk modulus of the oil was also calculated, and $Z_{SM,i}$ was substituted into the pump source flow ripple $Q_{S,i}$ calculation formula^26^. As shown in Fig. 30, the flow ripple amplitude and phase of the first 20 orders of the ripple frequency of the test pump were calculated. The time-domain curve of the flow ripple $q(t)$ at the working condition could be obtained by inverting the frequency-domain data, as shown in Fig. 31.

### Comparisons of theoretical, simulated, and experimental results

#### Average flow rate and volumetric efficiency

**Figure 32:**
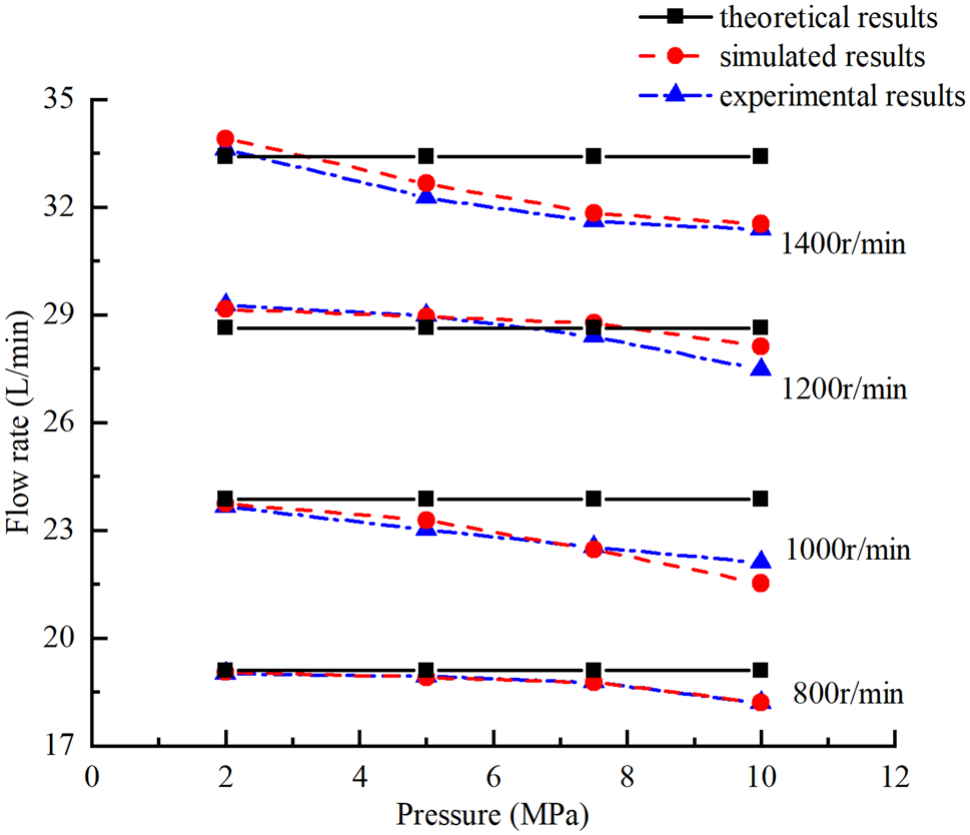
Comparisons of theoretical, numerically and experimentally obtained pressure–flow (p–Q) results.

**Figure 33:**
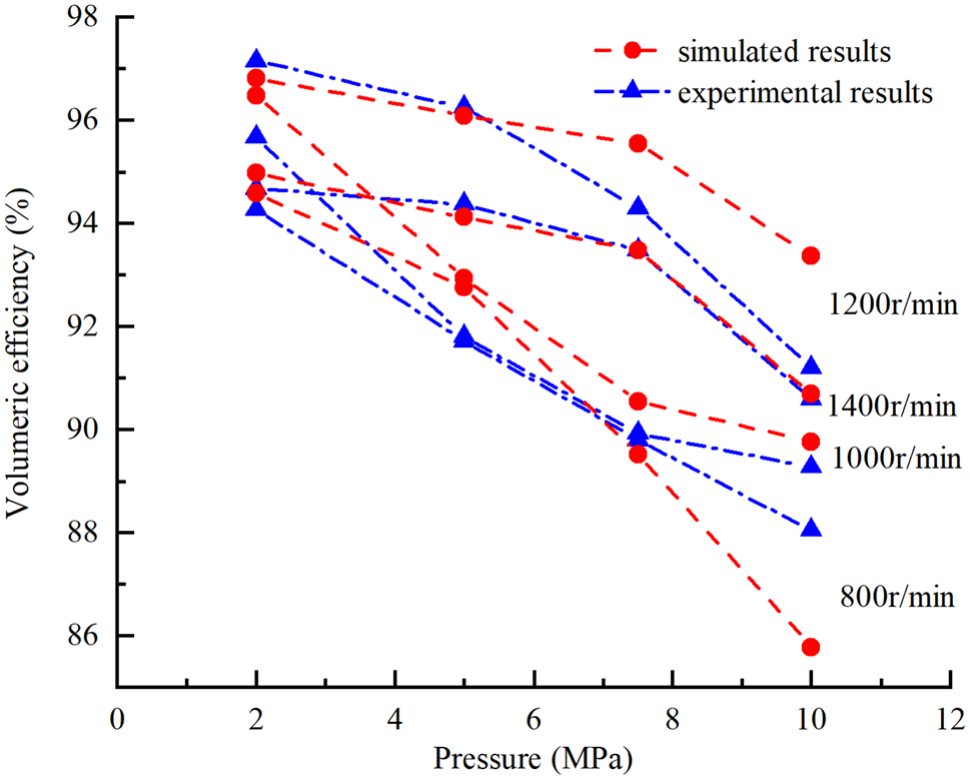
Comparisons of numerically and experimentally obtained volumetric efficiency curves.

The theoretically, numerically, and experimentally obtained pressure–flow (p-Q) results are compared in Fig. 32, the numerically, and experimentally obtained volumetric efficiency curves are shown in the Fig. 33. The following conclusions can be drawn:

1. With the increase in the outlet pressure from 2.0 to 10.0 MPa and the pump speed from 800 to 1400 r/min, the pressure–flow (p–Q) results and volumetric efficiency curves matched well for the simulated and experimental results. With the increase of pressure, the internal leakage of the pump increases, so the volume efficiency and output flow rate are lower, and the results show a downward trend. The accuracy and validity of the simulation model was verified.
2. The theoretical results were not in good agreement with the simulated and experimental results, and the deviation increased gradually as the pressure increased mainly because the internal leakage of the pump was not taken into account in the theoretical calculation.

#### Flow ripple

**Figure 34:**
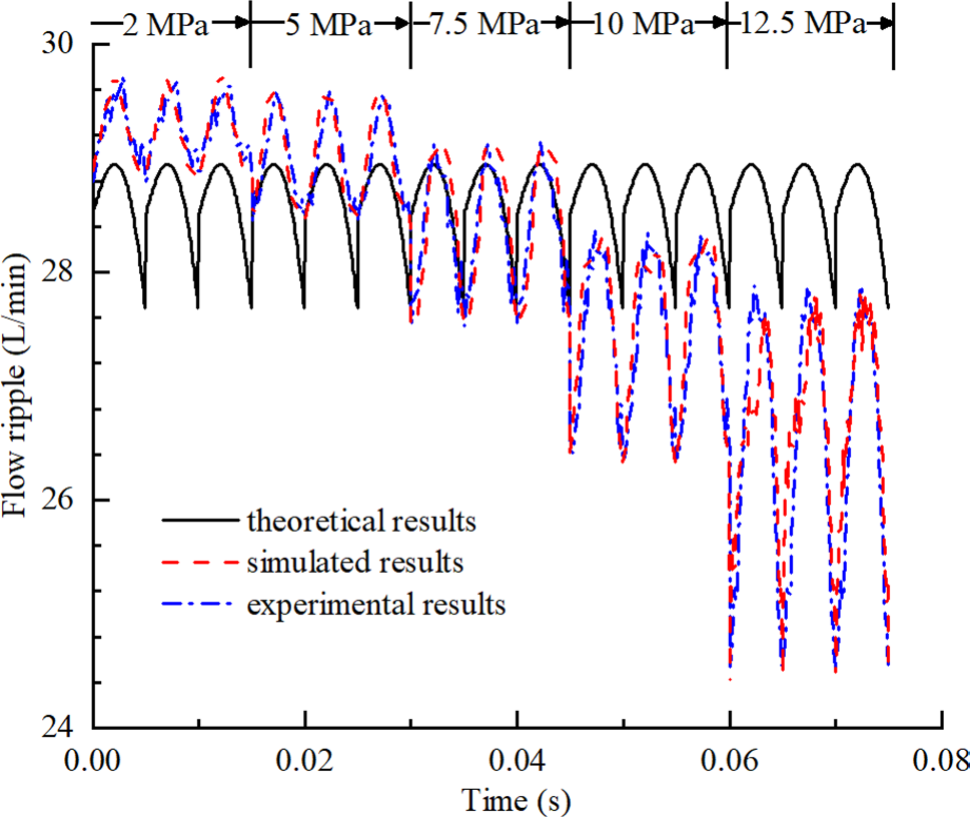
Comparison of flow ripple results under different outlet pressures ($n$ = 1200 r/min).

**Figure 35:**
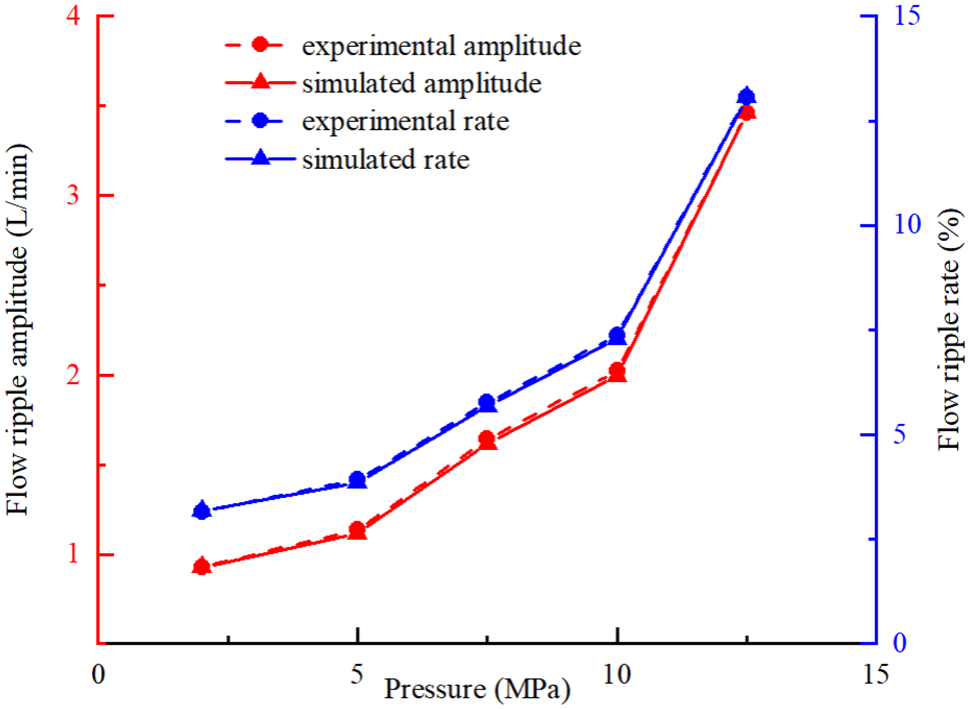
Influence of outlet pressure on flow ripple characteristics ($n$ = 1200 r/min).

**Figure 36:**
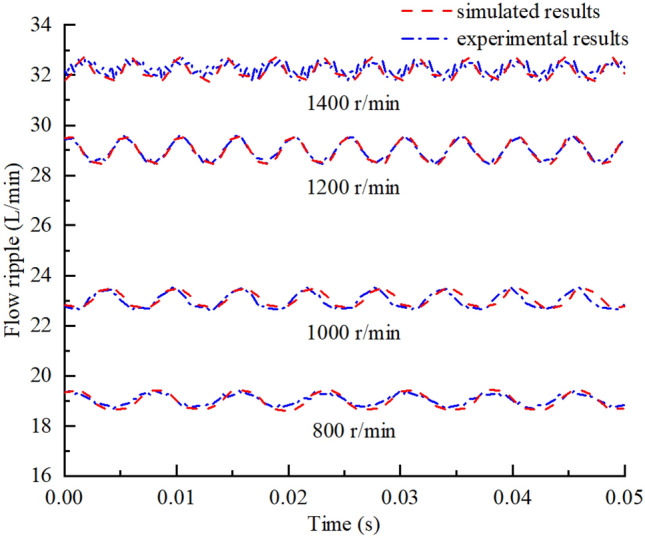
Comparison of flow ripple results under different pump speeds ($P_{0}$ = 5 MPa).

**Figure 37:**
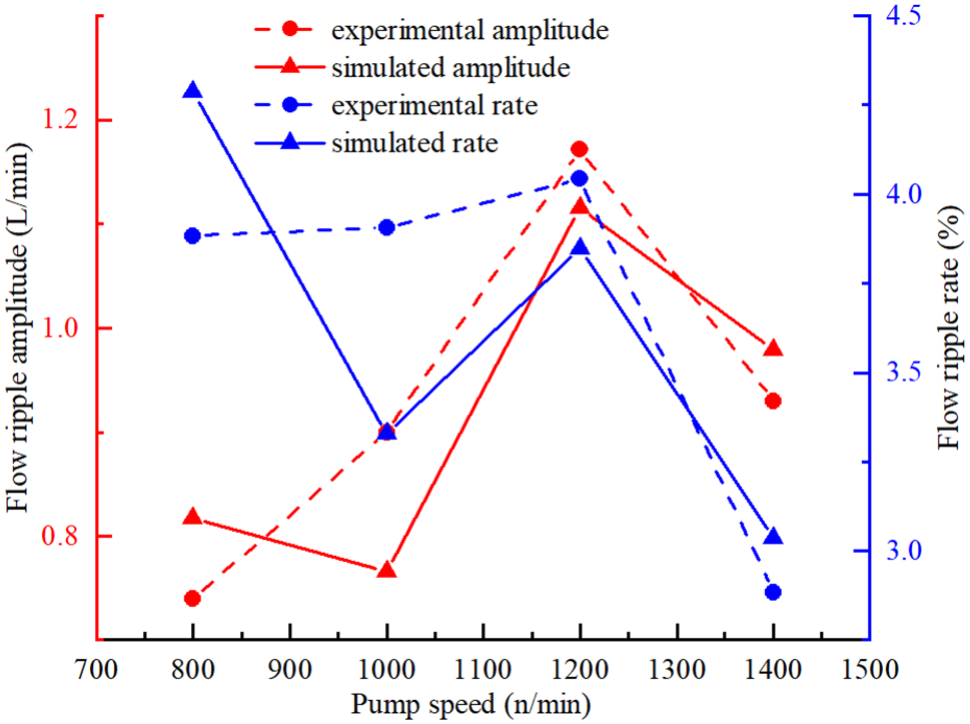
Influence of pump speed on flow ripple characteristics ($P_{0}$ = 5 MPa).

The theoretical, simulated, and experimental results for the flow ripple of the pump under different working conditions were compared to analyze the influence of the flow ripple characteristics. Comparisons of the flow ripple under different outlet pressures and the influence of the outlet pressure on the flow ripple characteristics are shown in Figs. 34 and 35, from which the following conclusions were obtained:

1. Because the compressibility of the oil and the internal leakage were neglected in the theoretical calculation and the influence of geometric structures such as unloading grooves were not taken into account, the agreement between the theoretical flow ripple curve and the simulation and experimental curves was poor, and the error increased gradually under high pressures. Thus, accurate predictions of the flow ripple cannot be achieved using the theoretical formula.
2. The consistency of the flow ripple curves between the simulation and test was good, and the comparison of theoretical and simulated results showed that flow ripple was the comprehensive result of the oil characteristics, internal leakage, and geometric characteristics.
3. With the increase in the outlet pressure, the flow ripple amplitude increased from 0.93 to 3.46 L/min, and the ripple rate increased from 3.17 to 13.07%. As the outlet pressure increased, the compressibility of the oil was greater and the backflow of the outlet lines increased. Thus, the nonuniformity of the flow rate increased, and the ripple amplitude increased. Meanwhile, the internal leakage flow of the pump increased, the average flow rate of the output decreased, and the ripple rate increased.
  The flow ripple results were compared at different pump speeds, and the influences of the pump speed on the flow ripple characteristics are shown in Figs. 36 and 37, from which we determined the following:
  1. As shown in Fig. 36, the higher the pump speed was, the smaller the flow ripple period became, and the more ripple waves appeared per unit time.
  2. The higher the speed was, the more likely the pump was to suck air in the test process, and the gas volume fraction in the pump increased. The gas volume fraction was assumed to be constant in the simulation, so there were certain errors between the simulation and experimental results.
  3. The influence of the speed change on the flow ripple characteristics was less than that of the pressure change. When the pump speed increased from 800 to 1400 r/min, the flow ripple amplitude varied from 0.73 to 1.17 L/min, and the flow rate varied from 2.88 to 4.29%.

## Conclusions

1. A theoretical flow ripple model for a Truninger pump was established using the vector ray method. The effects of the design parameters on the theoretical flow rate and the flow ripple characteristics were studied. The module should be as small as possible, the addendum coefficient and the half angle of the tooth profile should be as large as possible within the design specifications, and the pump speed should be reduced as much as possible during operation so that the vibration and noise level of the designed gear pump are lower.
2. A comprehensive three-dimensional simulation model was constructed in Simerics-MP+, which was validated by comparisons with experimental results obtained by the secondary source method. The compressibility of the oil, the internal leakage and the influence of geometric structures were not taken into account in the vector ray method, as a result, the consistency of theoretical and experimental results is poor, while the three-dimensional fluid simulation results and experimental results match well. The comparison between the two showed that flow ripple was the comprehensive result of the oil characteristics, internal leakage, and geometric characteristics, which must be considered during simulations. Furthermore, design guidelines can be obtained from the three-dimensional simulation results, which can be used to achieve low-noise designs and to optimize this type of pump.
3. The effects of the outlet pressure and pump speed on the flow ripple characteristics were studied. With the increase in the outlet pressure from 2.0 to 12.5 MPa, the flow ripple amplitude increased from 0.93 to 3.46 L/min, and the ripple rate increased from 3.17 to 13.07%. The results showed that the flow ripple amplitude and the ripple rate increased with the increase in the outlet pressure, which changed the oil properties, such as the compressibility. When the pump speed increased from 800 to 1400 r/min, the flow ripple amplitude varied from 0.73 to 1.17 L/min, and the flow rate varied from 2.88 to 4.29%. The influence of the pump speed variations on the flow ripple characteristics was less than that of outlet pressure variations, because the pump speed might change the gas volume fraction in the pump, which had a slight effect on the flow ripple characteristics.

## List of symbols

CAB: Conformal adaptative binary-tree
$\theta$: Central angle of the single tooth’s pitch circle
$\beta$: Half angle of the tooth profile
$$L$$: Perpendicular distance
$$N$$: Meshing point
$$P$$: Pitch point
$\rho_{1} ,\;\rho_{2}$,: Distance from the external gear center and the internal gear ring center to the meshing point, respectively
$\varphi$: Rotation angle of the external gear
$$a$$: Center distance
$r_{1} ,\;r_{2}$: Pitch radius of the external gear and the internal gear ring, respectively
$$i_{{12}}$$: Transmission ratio
$\omega$: Angular speed of the external gear
$$b$$: Tooth width
$r_{a1} ,\;r_{a2}$: Addendum circle radius of the external gear and the internal gear ring, respectively
$r_{f1} ,\;r_{f2}$: Dedendum circle radius of the external gear and the internal gear ring, respectively
$$m$$: Module
$z_{1}$: Number of external gear teeth
$z_{2}$: Number of internal gear ring teeth
$$h_{a}^{ * }$$: Addendum coefficient
$$h_{d}^{ * }$$: Dedendum coefficient
$q_{0}$: Oil discharge volume of a pair of teeth meshing
$q_{sh}$: Theoretical flow ripple
$q_{a}$: Flow ripple amplitude
$\delta_{q}$: Flow ripple rate
$q_{t}$: Average flow rate of the pump
$$n$$: Pump speed
$$\Omega$$: Control volume
$$\sigma$$: Surface normal
$\rho$: Average local fluid density
$$p$$: Static pressure
$$\overrightarrow {f}$$: Body force
$$\overrightarrow {\nu }$$: Fluid velocity
$$\overrightarrow {{\nu_{\sigma } }}$$: Mesh velocity
$\widetilde{\tau }$: Shear stress tensor
$$\mu$$: Fluid viscosity
$C_{\mu }$: Renormalization group (RNG) $k - \varepsilon$ turbulence model constant
$\eta_{0}$: RNG $k - \varepsilon$ turbulence model constant
$\beta_{0}$: RNG $k - \varepsilon$ turbulence model constant
$c_{1} ,\;c_{2}$: RNG $k - \varepsilon$ turbulence model constant
$k$: Turbulent kinetic energy
$\varepsilon$: Turbulent kinetic energy dissipation rate
$\sigma_{k}$: Turbulent Prandtl number of $k$
$\sigma_{\varepsilon }$: Turbulent Prandtl number of $\varepsilon$
$D_{f}$: Diffusivity of the vapor mass fraction
$\sigma_{t}$: Turbulent Schmidt number
$f_{v}$: Mass fraction of the vapour
$R_{e}$: Vapor generation term
$R_{c}$: Condensation rate term
$$\rho_{l}$$: Liquid density
$$\rho_{v}$$: Vapor density
$f_{g,f}$: Mass fraction of free non-condensable gases (NCGs)
$$C_{e}$$: Evaporation coefficient
$$C_{c}$$: Condensation coefficient
$f_{g,d}$: Mass fraction of dissolved NCGs
$D_{g,d}$: Diffusivity of dissolved NCGs
$\sigma_{g,d}$: Dissolved NCGs Schmidt number
$f_{g,d\;equil}$: Equilibrium mass fraction of the dissolved NCGs at the current cell pressure
$f_{d,equil,ref}$: Equilibrium mass fraction of the dissolved NCGs at the reference pressure
$$p_{d,equil,ref}$$: Reference pressure
$$T$$: Temperature
$$P_{in}$$: Inlet pressure
$$P_{out}$$: Outlet pressure
$Q_{S}$: Pump source flow ripple
$Z_{S}$: Pump source impedance
$Z_{SM}$: Source impedance in the full frequency band
$p_{1} \left( t \right),\;p_{2} (t)$: Time-domain data
$P_{1} \left( f \right),\;P_{2} (f)$: Frequency-domain data
